# The sugar composition of the fibre in selected plant foods modulates weaning infants’ gut microbiome composition and fermentation metabolites in vitro

**DOI:** 10.1038/s41598-021-88445-8

**Published:** 2021-04-29

**Authors:** Shanthi G. Parkar, Jovyn K. T. Frost, Doug Rosendale, Halina M. Stoklosinski, Carel M. H. Jobsis, Duncan I. Hedderley, Pramod Gopal

**Affiliations:** 1 The New Zealand Institute for Plant and Food Research Limited, Private Bag 11600, Palmerston North, 4442 New Zealand; 2 Riddet Institute, Massey University, Palmerston North, 4442 New Zealand

**Keywords:** Microbial ecology, Microbiome, Microbial communities, Microbiology

## Abstract

Eight plant-based foods: oat flour and pureed apple, blackcurrant, carrot, gold- and green-fleshed kiwifruit, pumpkin, sweetcorn, were pre-digested and fermented with pooled inocula of weaning infants’ faecal bacteria in an in vitro hindgut model. Inulin and water were included as controls. The pre-digested foods were analysed for digestion-resistant fibre-derived sugar composition and standardised to the same total fibre concentration prior to fermentation. The food-microbiome interactions were then characterised by measuring microbial acid and gas metabolites, microbial glycosidase activity and determining microbiome structure. At the physiologically relevant time of 10 h of fermentation, the xyloglucan-rich apple and blackcurrant favoured a propiogenic metabolic and microbiome profile with no measurable gas production. Glucose-rich, xyloglucan-poor pumpkin caused the greatest increases in lactate and acetate (indicative of high fermentability) commensurate with increased bifidobacteria. Glucose-rich, xyloglucan-poor oats and sweetcorn, and arabinogalactan-rich carrot also increased lactate and acetate, and were more stimulatory of clostridial families, which are indicative of increased microbial diversity and gut and immune health. Inulin favoured a probiotic-driven consortium, while water supported a proteolytic microbiome. This study shows that the fibre-derived sugar composition of complementary foods may shape infant gut microbiome structure and metabolic activity, at least in vitro.

## Introduction

The gut microbiota play an important role in host health, primarily because they are essential for metabolism of food, beginning with milk saccharides in infants to the more complex plant cell wall polysaccharides in the adult gut. The major microbial end-products of the glycan metabolism are organic acid (OA) metabolites which serve the host through nourishment of colonocytes, maintenance of gut barrier, inhibition of pathogens and promotion of anti-inflammatory processes^1^. Gut bacteria also play a major role in regulation of metabolism, absorption of iron, detoxification of drugs and the synthesis of vitamins and bioamines^1^.

The successive gut microbial colonisation in infants depends on the foods introduced, the time of introduction, infant health status, environment and exposure to antibiotics^2,3^. The newborn infant’s gut is sparsely populated mainly with, but not limited to, members of Proteobacteria and acquired in utero, during birth or from the environment^1,3,4^. The newborn gut microbiome shows vast inter-individual variability, driven largely by birth mode, then starts evolving within hours owing to maternal transmission, dependent mainly on the nature of the primary feed, i.e., breast milk or infant formula^4^. The neonatal gut is thus colonised by a founder group mainly from *Enterobacteriaceae*, *Bifidobacterium*, certain bacteria from *Bacteroidetes* and *Firmicutes*, with metagenome capacity largely focused on breaking down human milk oligosaccharides^1–3^.

Plant foods are commonly introduced (along with cheese and meat) to the infants at about 6 months of age, supporting the growth and development needs of the infants^5^. They are rich sources of plant cell wall polysaccharides, also known as dietary fibres, that provide the major source of energy for the bacteria in the growing child’s gut^1,6^. The human gut carbohydrases have mainly evolved to break down plant starches (1-4-α-glycosidic linkages) to glucose for absorption in the small intestine^7^, but not to degrade plant cell wall polysaccharides^8^. The major plant cell wall polysaccharides include cellulose and xyloglucan (found in peas and leafy greens), pectin (found in fruits and vegetables including apples, blackcurrants, carrots and kiwifruit), glucans (found in oats), mucilages (found in seeds), arabinogalactans (e.g. in wheat) and chitin (e.g. in mushroom)^9–11^. Whereas cellulose (1→4-β-linked glucose) and xyloglucan (1→4-β-linked glucose substituted with short xylose sidechains) have relatively simple structures, pectins are highly variable in their chemical composition and in their degree of branching^12,13^. Pectin has a backbone or smooth region composed mainly of homogalacturonan, or 1,4-linked α-D-galacturonic acid, occasionally substituted by rhamnose. Pectin’s “hairy” sidechains are called rhamnogalacturonans-I, and they consist of repeating units of (1,4)-galacturonosyl and α-(1,2)-rhamnosyl chains to which are attached glycans made up of neutral sugars, rhamnose and galactose^12^. This compositional and structural complexity of pectin has been suggested to stimulate the growth of different members of the gut bacterial community^12^.

Inulin, a plant fibre oligofructan isolate, is a well-known prebiotic, as it enhances the growth of beneficial bifidobacteria^14^. Other oligosaccharides, with varying sugar chemistries and structural complexities, have also been shown to selectively modulate infant gut bacteria or bacterial consortia that extract energy by producing specialised glycosidases which break down these oligosaccharides^15,16^.

While the prebiotic potential of isolated fibre fractions are quite well characterised, much less is known about the prebiotic capacity of whole food-based foods. Fruits and vegetables are known to be a source of structurally varied carbohydrates, with varying capacities to alter different gut microbiota^5,17^. Exploiting the natural diversity of plant cell wall polysaccharides may be an attractive option to formulate infant complementary foods with improved functional benefits to gut health.

In this study, we hypothesised that foods derived from fruits, vegetables and a grain will vary in their capacity to modulate different members of the gut microbial community and their functional and metabolic capacities. The objective of this study was to establish the impact of food groups on the infant gut microbiome by incubation of pre-digested foods (under simulated conditions of the foregut) with infant faecal bacteria. The food-microbiome interactions in the fermentation were then characterised by examining the relationships between the plant-food fibre-derived carbohydrates and the microbial response to the plant-food (microbial glycosidase analysis), microbial metabolism (acid and gas production) and microbiome characterisation using 16S rRNA gene sequencing.

## Results

### Fibre-derived sugar composition

**Figure 1:**
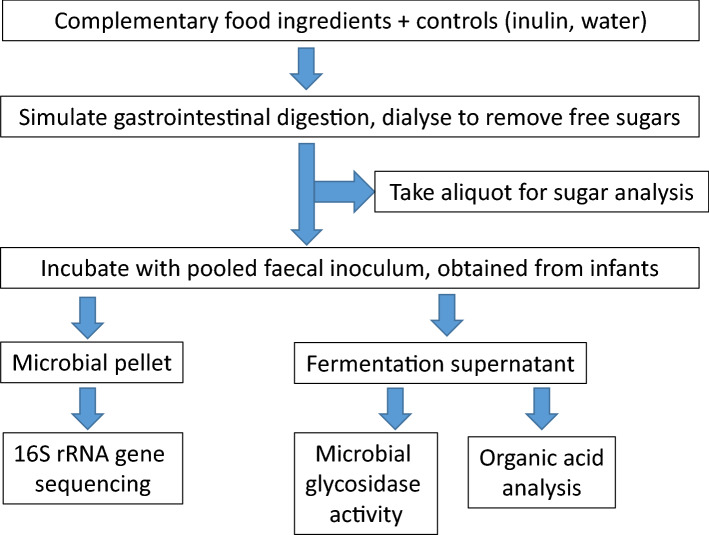
Workflow of the experimental protocol and analyses.

Eight foods (as given in Table 1), inulin as a positive control, and water (digesta control) were digested in vitro (as outlined in Fig. 1) and the non-cellulosic neutral sugar and uronic acid composition of the foods were analysed after digestion (Table 2). The concentration of glucose was highest in oats, pumpkin and sweetcorn, at 387, 231, and 185 µg/mg, respectively. The fruits (apple, blackcurrant and kiwifruit) also contained relatively high concentrations of glucose (94–112 µg/mg) but were also accompanied by higher amounts of xylose (12–14 µg/mg). The fruits therefore show a higher xylose/glucose ratio (between 0.17 and 0.14 with apple > blackcurrant > green-fleshed kiwifruit > gold-fleshed kiwifruit) compared with the vegetables and cereal (ratio between 0.07 and 0.02, sweetcorn > carrot > oats > pumpkin).

**Table 1:** Foods used for the in vitro gastro-intestinal digestion and fermentation.

Food	Supplier
Apple puree	Frupak, Hastings, New Zealand
Blackcurrant puree, seedless	Juice Products New Zealand, Timaru, New Zealand
Carrot puree	Juice Products New Zealand, Timaru, New Zealand
Gold-fleshed kiwifruit puree, seed-out	Kiwifruitz, Tauranga, New Zealand
Green-fleshed kiwifruit puree, seed-out	Kiwifruitz, Tauranga, New Zealand
Oat flour	Harraway & Sons Ltd., Dunedin, New Zealand
Pumpkin puree	Cedenco Foods New Zealand Ltd., Auckland, New Zealand
Sweetcorn puree	Cedenco Foods New Zealand Ltd., Auckland, New Zealand

**Table 2:** Neutral sugar and uronic acid composition (µg/mg) and molar ratio analysis of pectic and xyloglucan-related sugars in the digesta from foods and inulin.

Sugar	Apple	Blackcurrant	Carrot	Gold-fleshed kiwifruit	Green-fleshed kiwifruit	Inulin	Oats	Pumpkin	Sweet corn
Rhamnose	6. ± 0.4	14.6 ± 0.5	10.1 ± 0.6	4.1 ± 0.1	4.4 ± 0.2	0.6 ± 0.0	0.4 ± 0.0	4.6 ± 0.1	1.4 ± 0.1
Fucose	3.6 ± 0.3	1.2 ± 0.0	1.0 ± 0.1	1.1 ± 0.0	1.4 ± 0.1	0.8 ± 0.0	0.2 ± 0.0	0.6 ± 0.0	0.2 ± 0.1
Arabinose	22.5 ± 1.4	12.3 ± 0.4	29.8 ± 1.1	6.6 ± 0.5	7.4 ± 0.0	3.5 ± 0.0	10.7 ± 1.9	9.7 ± 0.1	14.8 ± 0.3
Xylose	13.9 ± 1.7	12.2 ± 0.2	3.8 ± 0.1	11.8 ± 0.8	13.3 ± 0.2	0.5 ± 0.1	8.9 ± 2.6	4.6 ± 0.5	11.5 ± 0.2
Mannose	5.5 ± 0.1	4.9 ± 0.0	6.9 ± 0.4	6.8 ± 0.4	6.1 ± 0.1	5.0 ± 0.1	3.1 ± 0.5	5.2 ± 0.2	2.3 ± 0.1
Galactose	20.9 ± 1.1	17.8 ± 0.5	52.6 ± 2.9	24.1 ± 1.7	26.3 ± 0.2	17.3 ± 0.1	10.8 ± 0.6	32.5 ± 0.1	14.6 ± 0.2
Glucose	100.0 ± 1.2	94.0 ± 3.3	96.1 ± 8.0	112.3 ± 6.2	111.8 ± 6.9	62.6 ± 0.3	387.1 ± 14.6	231.0 ± 24.2	185.1 ± 0.6
Uronic acid	124.7 ± 11.3	111.2 ± 5.5	130.7 ± 13.6	96.8 ± 3.3	113.2 ± 4.7	19.7 ± 0.9	24.4 ± 1.7	70.3 ± 7.7	39.7 ± 2.0
Total	297.5 ± 6.2	268.2 ± 5.0	331.0 ± 12.9	263.6 ± 2.9	283.8 ± 6.8	110.0 ± 0.5	445.6 ± 19.0	358.5 ± 23.9	269.6 ± 0.5
**Molar ratio**									
Uronic acid/rhamnose	15.0	5.9	10.0	18.3	19.7	24.2	42.4	11.9	22.0
(Arabinose + Galactose)/ Rhamnose	6.8	2.0	8.0	7.2	7.2	31.1	48.4	8.8	21.1
Xylose/Glucose	0.17	0.16	0.04	0.14	0.16	0.01	0.03	0.02	0.07

Values (µg/mg of the freeze-dried material) are mean of two determinations, measured in duplicate. S.E. shown.

The amount of uronic acid was highest in carrot, apple, blackcurrant and kiwifruit, ranging from 97 to 131 µg/mg, while the amount of uronic acid was lower in oats, pumpkin and sweetcorn, ranging from 24 to 70 µg/mg. Rhamnose was highest in blackcurrant, at 15 µg/mg, while arabinose and galactose were highest in carrot, at 30 and 53 µg/mg, respectively. Apple had the highest amount of fucose (3.56 µg/mg), while carrot followed by both kiwifruit had the highest amount of mannose (> 6 µg/mg). The fruits and carrot had higher molar proportions of uronic acid (32–37%) than oats, pumpkin and sweetcorn (Supplementary Table S2 online). Comparing the uronic acid-rich foods, blackcurrant had the lowest uronic acid/rhamnose ratio at 5.9, while green-fleshed kiwifruit had the highest, at 19.7. Blackcurrant also had the lowest (arabinose + galactose)/rhamnose ratio, at 2.0, while carrot had the highest, at 8.0.

### Headspace gas measurement

Increases of head space gas pressure were noted from the 5-h sampling time for carrot, oats and sweetcorn, and the controls, i.e., water digesta and inulin (Table 3). Apple, blackcurrant, green-fleshed or gold-fleshed kiwifruit and pumpkin did not cause any measurable increase in headspace gas. Whilst fermentative gas pressures continued to rise in the control fermenta, food gas pressures peaked at 10 h and declined thereafter.

**Table 3:** Gas production (headspace PSI) during fermentation of substrates.

Time (h)	Apple	Blackcurrant	Carrot	Digesta control	Gold-fleshed kiwifruit	Green-fleshed kiwifruit	Inulin	Oats	Pumpkin	Sweetcorn
0	0.00 ± 0.00	0.00 ± 0.00	0.00 ± 0.00	0.00 ± 0.00	0.00 ± 0.00	0.00 ± 0.00	0.00 ± 0.00	0.00 ± 0.00	0.00 ± 0.00	0.00 ± 0.00
5	0.00 ± 0.00^a^	0.00 ± 0.00^a^	0.50 ± 0.25^ab^	0.50 ± 0.43^ab^	0.00 ± 0.00^a^	0.00 ± 0.00^a^	0.75 ± 0.00^b^	0.83 ± 0.29^b^	0.00 ± 0.00^a^	0.67 ± 0.14^b^
10	0.00 ± 0.00^a^	0.00 ± 0.00^a^	0.83 ± 0.14^b^	1.67 ± 0.14^c^	0.00 ± 0.00^a^	0.00 ± 0.00^a^	1.33 ± 0.29^c^	0.83 ± 0.14^b^	0.00 ± 0.00^a^	0.67 ± 0.14^b^
16	0.00 ± 0.00^a^	0.00 ± 0.00^a^	0.00 ± 0.00^a^	3.83 ± 0.14^b^	0.00 ± 0.00^a^	0.00 ± 0.00^a^	3.75 ± 0.25^b^	0.25 ± 0.43^a^	0.00 ± 0.00^a^	0.50 ± 0.43^a^
24	0.00 ± 0.00^a^	0.00 ± 0.00^a^	0.00 ± 0.00^a^	2.58 ± 0.14^b^	0.00 ± 0.00^a^	0.00 ± 0.00^a^	2.42 ± 0.14^b^	0.00 ± 0.00^a^	0.00 ± 0.00^a^	0.00 ± 0.00^a^

Values are mean of three replicates. S.E. shown. At each time point, the significance due to the substrate was* P* < 0.001. Within a time point, means with letters in common are not significantly different. Tukey’s HSD (0.05).

### Organic acid analysis

The organic acid (OA) microbial metabolites were measured at 0, 5, 10, 16 and 24 h. At 0 h, all acids were below or around our limit of detection (Supplementary Table S3 online). Throughout the course of fermentation, the only acids to show significant changes (*P* < 0.001) in concentration were formate, lactate, acetate and propionate (Supplementary Table S4 online). Butyrate changes were significant between substrates (*P* < 0.001) only at 24 h. Formate generally increased from 5 h and decreased after 16 h; acetate and lactate increased from 5 h and continued increasing throughout the fermentation; while changes in propionate were predominant at 16 and 24 h (Supplementary Table S4 and Figure S1 online). Isobutyrate, isovalerate, valerate, hexanoate, heptanoate and succinate values were determined but are not shown, as they remained around or below the limit of detection.

**Figure 2:**
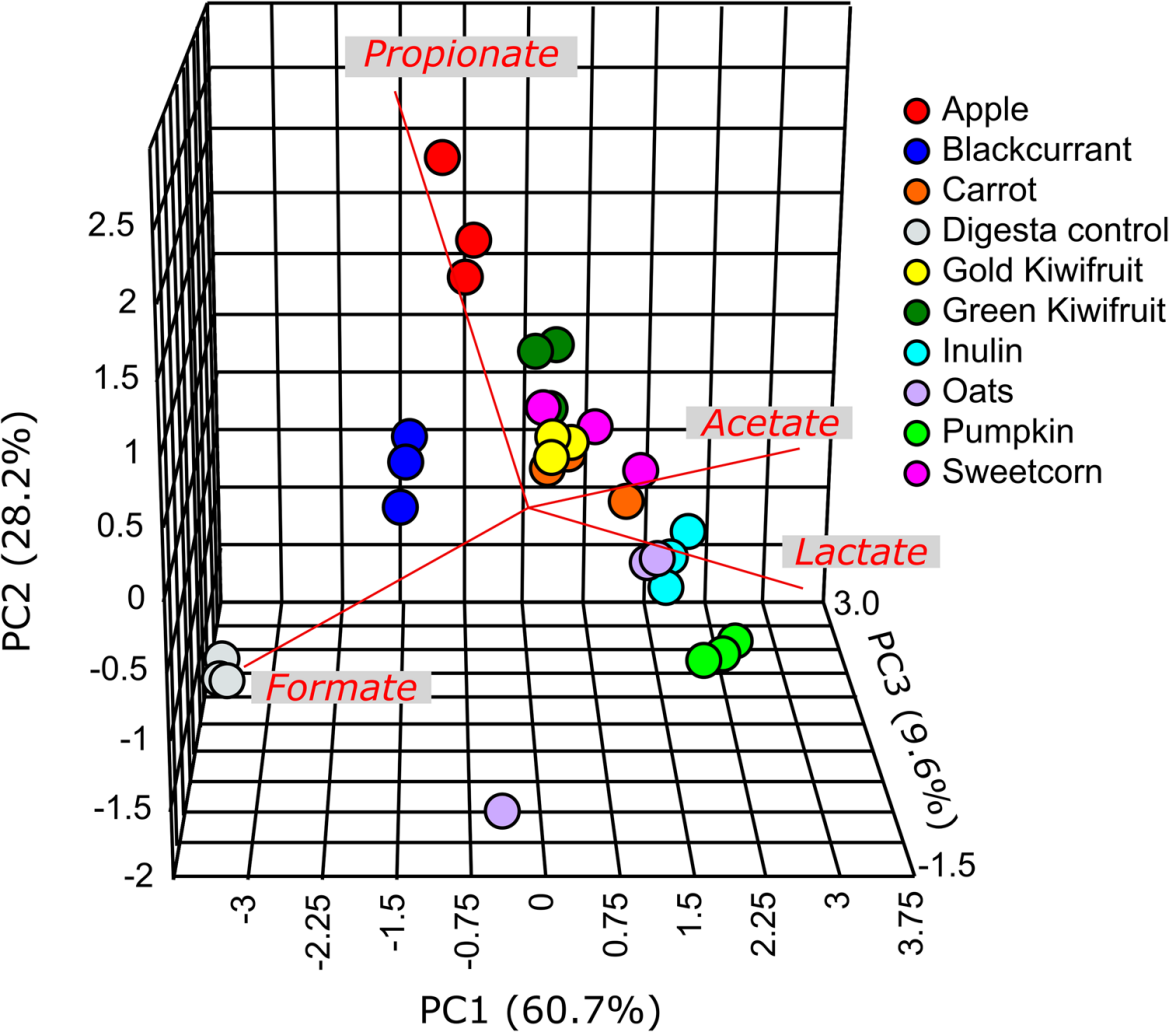
Principal component analysis plot visualising the changes in the organic acids after 10 h of fermentation of the foods and the two controls, inulin and water digesta. The acids that influence the loading are appended to the plot in red.

As seen in the PCA plot (Fig. 2), the acid profiles were found to discriminate between substrates at the end of 10 h, the time point when the gas production was also the greatest. The apple and blackcurrant fermenta OA profiles clustered farthest from that of pumpkin fermenta. Of the remaining foods, green-fleshed and gold-fleshed kiwifruit profiles clustered together, followed by those of carrot, sweetcorn and oats.

The digesta control separated from the other substrates in having the lowest lactate, acetate, and highest formate concentration. Inulin fermenta had the second highest formate concentration (~ 2-fold that of apple), with the profile clustering between those of oats and pumpkin fermenta. Of the foods at 10 h of fermentation, formate was the lowest in oats and highest in apple fermenta. Lactate was lowest in the fermenta from blackcurrant (the same value as in digesta control, i.e., 0.3 µM/mL) and highest in that from pumpkin (8.7 µM/mL), and of a similar range in fermenta from oats, sweetcorn and carrot (average values between 5.0 and 5.2 µM/mL). The acetate was lowest for blackcurrant (9.6 µM/mL) and highest for pumpkin (13.8 µM/mL) and between 11.9 and 12.7 µM/mL for the remaining foods. Propionate was the highest in apple fermenta (3.2 µM/mL) and lowest in pumpkin fermenta (1.6 µM/mL), followed very closely by oats fermenta (1.7 µM/mL). Blackcurrant fermenta thus had the highest formate and lowest lactate and acetate.

### Microbial glycosidase activity

**Figure 3:**
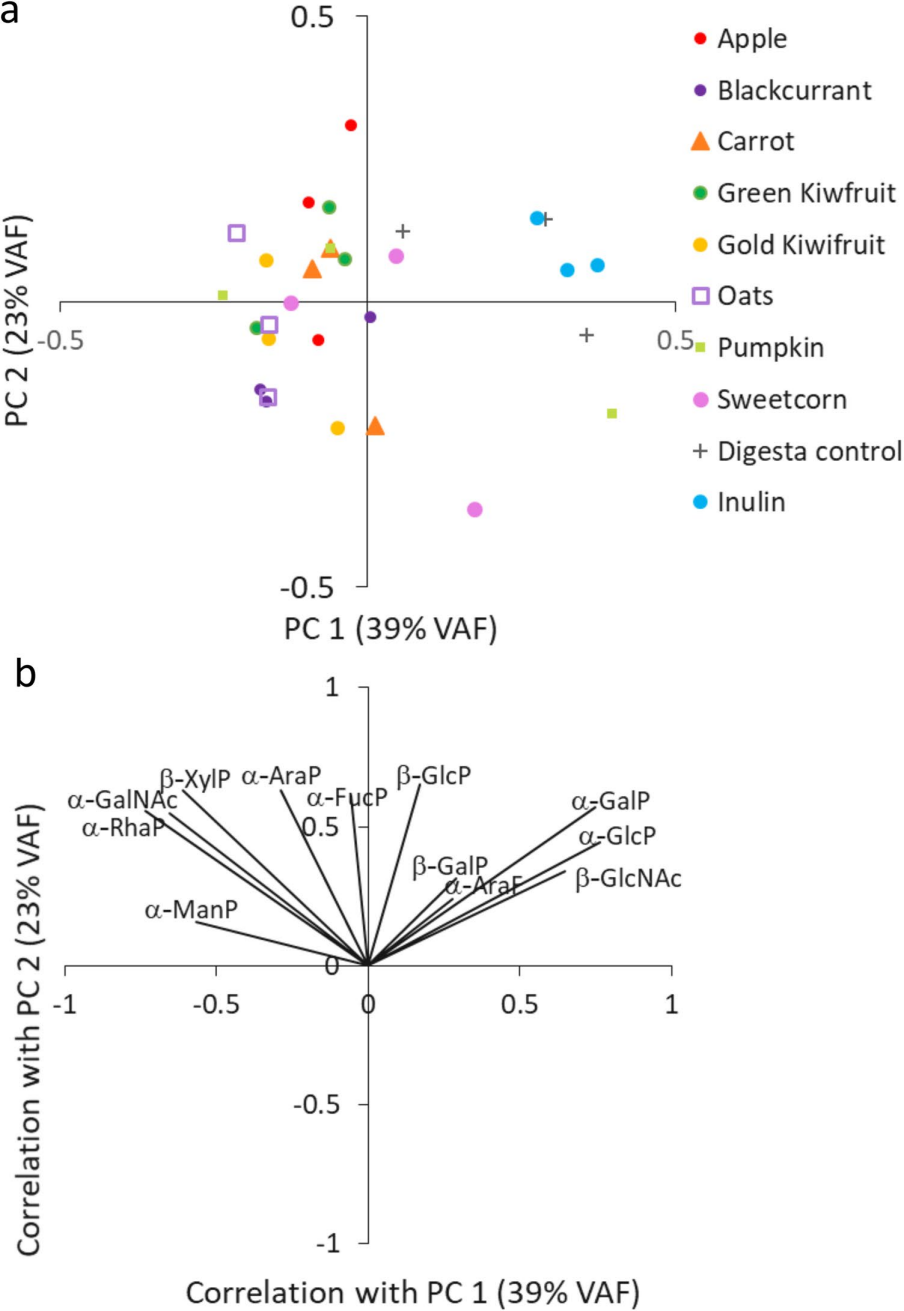
Principal component analysis plot (**a**) and the loadings plot (**b**) demonstrate microbial glycosidase-driven separation of foods and controls after 10 h of fermentation with infants’ faecal inocula. Each substrate was fermented in triplicate. The enzymes included α-arabinofuranidase (α-AraF), α-arabinopyranosidase (α-AraP), α-fucopyranosidase (α-FucP); α-glucopyranosidase (α-GlcP), β-glucopyranosidase (β-GlucoseP), α- and β-galactopyranosidase (β-GalP), α-N-acetylgalactosaminidase (GalNAc), β-N-acetylglucosaminidase (β-GlcNAc), α-rhamnopyranosidase (α-RhaP), α-mannopyranosidase (α-ManP) and β-xylopyranosidase (β-XylP).

In terms of relative microbial glycosidase activities (10 h versus 0 h) (Supplementary Table S5 online), the most consistent increases were observed with apple and gold-fleshed kiwifruit, and most consistent decreases were with blackcurrant and green-fleshed kiwifruit. Alpha galactosidases, –mannosidases and–rhamnosidases were the most commonly elevated enzymes, but with no statistically significant differences between the treatments. The PCA plot (Fig. 3) depicts the relative changes in the microbial glycosidases due to the foods and controls and shows that the inulin and digesta controls separated because of increased α-glucosidase and α-galactosidase activity. All the foods showed increases of different glycosidases, with least increases being seen with gold-fleshed kiwifruit, blackcurrant, oats and pumpkin.

### Microbiome characterisation

**Figure 4:**
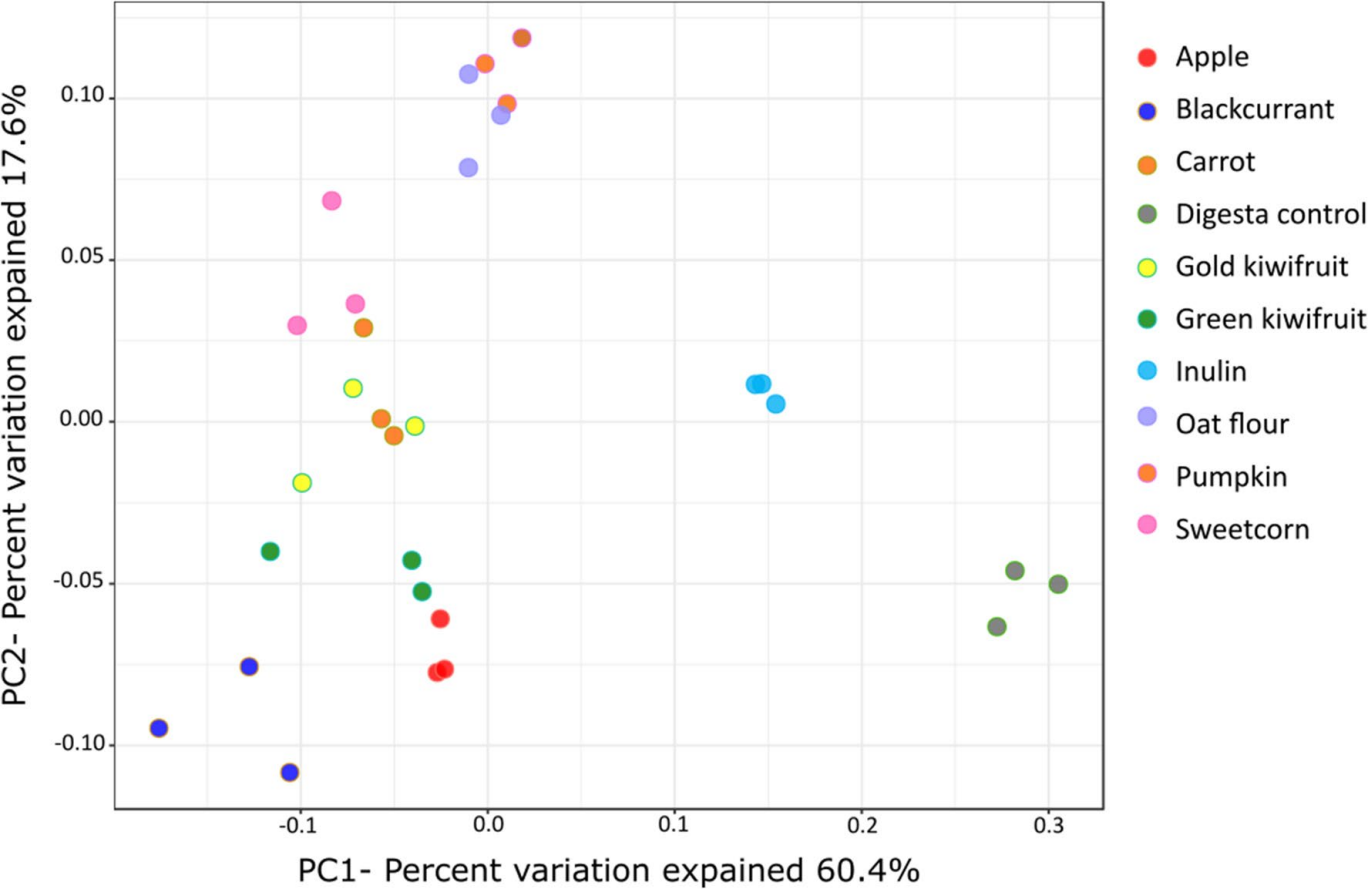
Bray–Curtis similarity index-based Principal Coordinate Analysis plot of the infant gut microbiome demonstrating the shifts in the community structure after 10 h of fermentation of the different foods in comparison to the no-carbohydrate digesta control. Each fermentation was carried out in triplicate.

After the 16S rRNA gene sequencing analysis for 0-h, 5-h and 10-h fermenta DNA, a total of 8,004,878 reads were obtained. There were only 107 to 198 reads for the three 5-h inulin fermenta DNA, and these were not used for analysis. With the remaining samples, a minimum of 35,002 reads per sample and a maximum of 250,729 reads were observed. Diversity analysis showed a significant effect of duration of fermentation (data not shown). The α-diversity decreased from 0 h to 10 h (*P* < 0.001, Shannon index). In terms of β-diversity, microbiome communities of the different time points showed separate clusters, and this spread increased with time, and the top three influencers were *Veillonella*, *Bifidobacterium* and *Enterobacteriaceae;g_* (*P* < 0.001, Bray-Curtis distance, data not shown). Examining the diversity trend at the end of 10 h fermentation separately, the α-diversity was significantly affected by the substrate (*P* = 0.003, Shannon index, supplementary Figure S2 online), with blackcurrant showing the least diversity. The digesta control showed the highest α-diversity, followed by inulin, carrot and apple. The Bray-Curtis plot visualising the β-diversity showed significant differences (*P* < 0.001), with the blackcurrant, pumpkin and digesta control microbial clusters being the most dissimilar from each other (Fig. 4).

**Figure 5:**
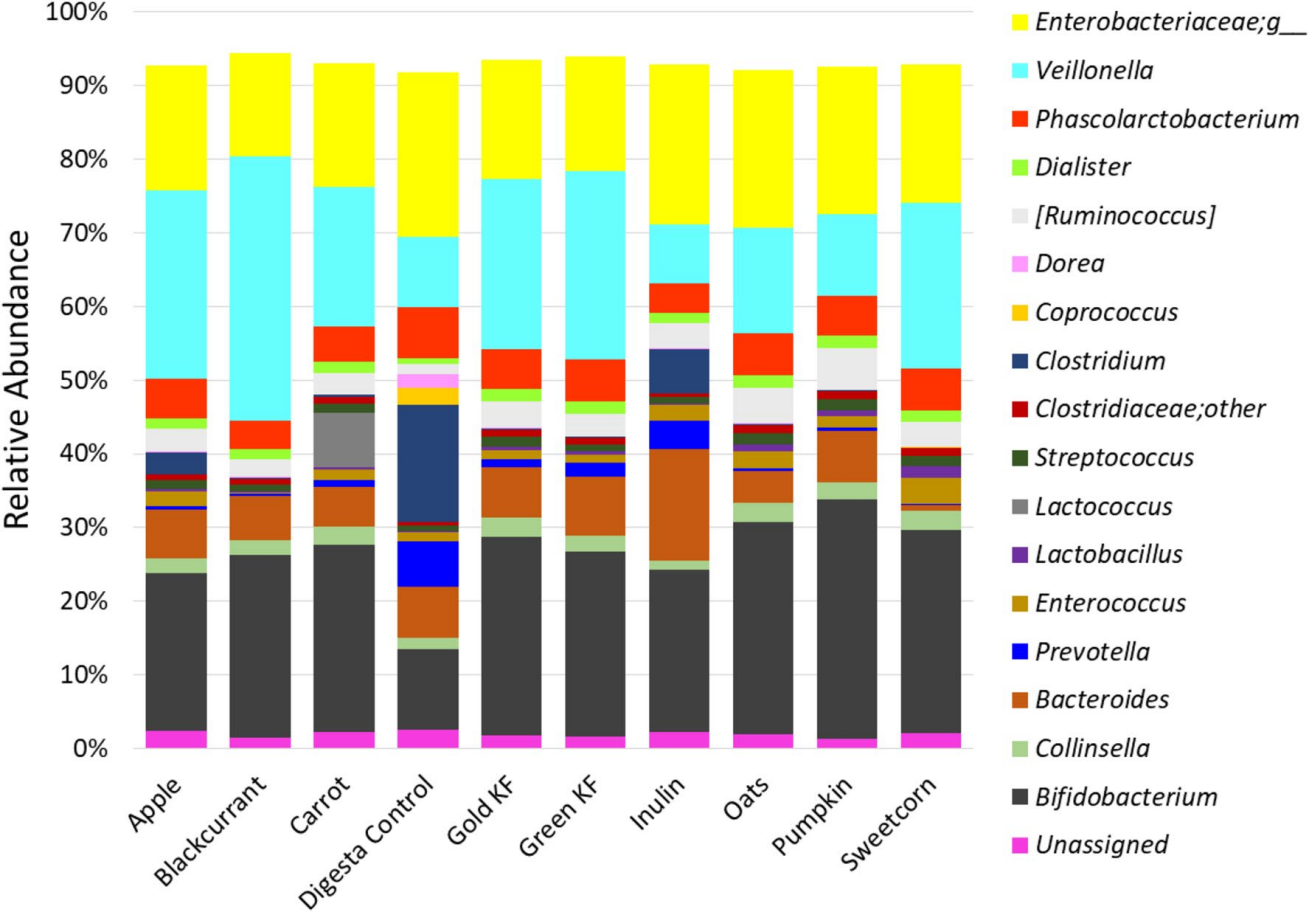
Significant changes in relative abundances in the infant faecal bacteria at the end of 10 h of fermentation. Genera/taxa with ≥ 1% relative abundance in at least one sample depicted. The significance values adjusted to the false discovery rate were *P* < 0.005, the likelihood ratio test was adjusted for 32 tests.

The pooled 0-h inoculum showed an average relative abundance (RA) of four major phyla: 21.4% *Actinobacteria*, 29.0% *Bacteroidetes*, 38.5% *Firmicutes* and 9.6% *Proteobacteria* (Table S6). At the level of genus or the nearest identifiable taxonomical level, some of the bacteria were *Bifidobacterium* (16.9% RA), *Bacteroides* (19.6% RA), *Phascolarctobacterium* (2.1% RA), *Pseudoramibacter_Eubacterium* (5.1% RA), and *Prevotella*, *Veillonella* and *Enterobacteriaceae;g_*at about 8% RA each, with many *Firmicutes* genera being < 1% RA (Table S7). The bacterial taxa showed significant changes at the end of 5 h and this trend was amplified at the end of 10 h of fermentation, as given in supplementary Tables S8 and S9 online, respectively. The 10-h microbiome showed a greater separation of foods in terms of microbiota changes and is depicted in Fig. 5 as changes in RAs, to better visualise substrate-driven microbiome changes. The digesta control had the lowest RA of *Bifidobacterium* and increased both *Bacteroidetes* (highest RA of *Parabacteroides* and *Prevotella*) and *Firmicutes* (highest RA of *Clostridium*, *Coprococcus, Dorea* and *Oscillospira*). Besides the expected increase in *Bifidobacterium* (21.9% RA), inulin caused the highest decrease in *Collinsella*, *Streptococcus* and *Clostridiaceae;other*, *Pseudoramibacter_Eubacterium*, *Phascolarctobacterium*, and highest increase in *Bacteroides* (15.1% RA)*.*

Some of the unique (and significant) changes in RA noted with different foods were as follows. After the digesta control, apple had the least *Bifidobacterium* (21.4% RA), with moderate increases in *Bacteroides* and changes in *Firmicutes* mainly driven by increases in *Veillonella.* Blackcurrant drove the inhibition of *Enterococcus* (0.3% RA compared with 3.5% RA for sweetcorn), *Lactobacillus* (0.1% compared with 1.6% RA for sweetcorn), *Phascolarctobacterium* (3.9% RA) and *Enterobacteriaceae;g__* (14.1% RA compared with > 21% RA in oats and the controls), while causing maximum increase in *Veillonella* (35.9% RA compared with 11.1% for oats). The kiwifruit appeared to have an intermediate response in terms of stimulation of gut bacteria. They were more bifidogenic than apple and blackcurrant, but less so than pumpkin. Green-fleshed kiwifruit caused the greatest increase in *Bacteroides* (8.0% RA compared with 0.8% RA in sweetcorn). While they were mainly suppressive towards most *Firmicutes* genera, gold-fleshed kiwifruit did increase *[Ruminococcus]* (3.6% RA), although not as much as pumpkin did. Pumpkin caused maximum increase in *Bifidobacterium* (32.5% RA), *[Ruminococcus]* (5.6% RA) and decrease in *Megasphaera*. Sweetcorn caused the maximum decrease in all the major Bacteroidetes genera (*Bacteroides*, *Prevotella* and *Parabacteroides*). This, along with the maximum increase in *Lactobacillus*, *Enterococcus*, *Ruminococcaceae;g__* and *Faecalibacterium* with moderate increase in *Veillonella* in sweetcorn 10-h fermenta, caused a large bloom in *Firmicutes*. Carrot was the only food to increase *Lactococcus* (7.3% RA versus the < 0.01% RA for all other treatments). In terms of common patterns of microbiome changes amidst the foods, pumpkin and oats caused similar stimulatory effects on *Streptococcus* and *Clostridiaceae;other* and inhibitory effects on *Veillonella*. The fruits (green-fleshed kiwifruit > apple > gold-fleshed kiwifruit ≥ blackcurrant, in order of inhibitory activity) were more inhibitory of the family *Ruminococcaceae*, while carrot, sweetcorn and oats (in that order) were stimulatory. Blackcurrant, green-fleshed kiwifruit and carrot (in that order) were more inhibitory of *Lachnospiraceae*, while pumpkin and oats were stimulatory.

Hypothesis testing using Spearman’s correlation coefficient revealed significant correlations (FDR-adjusted *P* < 0.10) between fibre-derived sugar composition of the foods and the 10-h microbiome abundances, depicted in Table 4. Glucose showed strong correlations (*r* = 0.9) with *Bifidobacterium*, *Lactobacillus, Streptococcus* and *[Ruminococcus].* The molar % of fucose in the foods inversely correlated with both *Clostridiaceae;other* and *Faecalibacterium* (*r* = − 0.9). *Phascolarctobacterium* correlated negatively with both the absolute concentrations and molar % of rhamnose (*r* = − 0.9) and positively (*r* = 0.9) with the uronic acid/rhamnose and (arabinose + galactose)/rhamnose ratio. The increase in xylose/glucose ratio tended to decrease most bacterial abundances, but showed strong positive correlations with *Veillonella* (*r* = 0.9).

**Table 4:** Spearman correlations between fibre-derived sugar composition of the foods and the changes in bacterial taxa at the end of 10 h of fermentation.

Concentration		Molar percentage					
Rhamnose	Glucose	Rhamnose	Fucose	Uronic acid/ Rhamnose	(Arabinose + Galactose)/ Rhamnose	Xylose/ Glucose	Organism
− 0.25	**0.90**	− 0.42	− 0.88	0.10	0.35	− 0.41	*Bifidobacterium*
− 0.38	0.78	− 0.41	− 0.80	0.30	0.31	− 0.22	*Collinsella*
− 0.72	0.18	− 0.78	− 0.17	0.72	0.77	− 0.51	*Eggerthella*
0.10	− 0.18	0.11	0.57	− 0.10	− 0.05	− 0.05	*Bacteroides*
− 0.03	− 0.47	0.11	0.81	0.07	− 0.03	0.06	*Parabacteroides*
− 0.13	− 0.43	0.04	0.64	0.17	− 0.01	− 0.26	*Prevotella*
− 0.40	0.67	− 0.54	− 0.54	0.38	0.45	− 0.21	*Enterococcus*
− 0.42	**0.93**	− 0.54	− 0.69	0.35	0.46	− 0.07	*Lactobacillus*
− 0.28	0.53	− 0.27	− 0.64	0.25	0.16	− 0.45	*Lactococcus*
− 0.32	**0.92**	− 0.43	− 0.76	0.18	0.36	− 0.37	*Streptococcus*
− 0.18	0.85	− 0.38	− **0.90**	0.05	0.30	− 0.47	*Clostridiaceae;Other*
− 0.62	0.22	− 0.75	− 0.28	0.55	0.76	− 0.63	*Clostridiaceae;g__*
0.07	− 0.68	0.17	0.66	0.05	− 0.18	0.11	*Clostridium*
− 0.63	0.22	− 0.72	− 0.36	0.53	0.71	− 0.89	*Pseudoramibacter_* *Eubacterium*
− 0.45	0.65	− 0.68	− 0.50	0.38	0.64	− 0.51	*Lachnospiraceae;g__*
0.35	0.43	0.17	− 0.38	− 0.42	− 0.16	− 0.05	*Blautia*
0.35	− 0.88	0.48	0.82	− 0.25	− 0.39	0.24	*Coprococcus*
0.27	− 0.53	0.18	0.29	− 0.32	− 0.09	− 0.13	*Dorea*
− 0.30	**0.95**	− 0.44	− 0.59	0.18	0.39	− 0.20	*[Ruminococcus]*
0.05	0.50	− 0.07	− 0.83	− 0.15	− 0.03	− 0.32	*Ruminococcaceae;g__*
− 0.15	0.67	− 0.28	− **0.91**	0.05	0.19	− 0.26	*Faecalibacterium*
− 0.12	− 0.48	− 0.06	0.64	0.22	0.12	0.49	*Oscillospira*
− 0.23	0.88	− 0.37	− 0.82	0.07	0.31	− 0.41	*Ruminococcus*
− 0.15	0.78	− 0.24	− 0.43	0.07	0.19	− 0.22	*Dialister*
0.18	− 0.18	0.35	0.47	− 0.02	− 0.39	0.65	*Megasphaera*
− **0.93**	0.32	− **0.94**	− 0.19	**0.93**	**0.95**	− 0.52	*Phascolarctobacterium*
0.58	− 0.22	0.70	0.36	− 0.47	− 0.69	**0.94**	*Veillonella*
− 0.62	0.62	− 0.81	− 0.55	0.55	0.77	− 0.62	*Erysipelotrichaceae;g__*
0.23	0.10	0.38	− 0.19	− 0.28	− 0.40	0.44	*Sutterella*
− 0.55	0.05	− 0.59	0.08	0.55	0.61	− 0.25	*Enterobacteriaceae;* *Other*
− 0.68	0.27	− 0.81	− 0.25	0.63	0.81	− 0.68	*Enterobacteriaceae;g__*

Only sugars with at least one correlation (*r* ≥ ± 0.9 shown in bold, FDR-adjusted *P* < 0.1, multiple hypothesis testing) are presented.

## Discussion

Eight commercially available foods, along with two controls, water and inulin, were subjected to an in vitro enzymatic digestion protocol followed by dialysis, as a simulation of infant upper gut digestion. The digested materials, which varied in their sugar composition, were then fermented with infant faecal bacteria under anaerobic conditions for up to 24 h. The purpose of this exercise was to develop understanding of the impact of commonly available fruit and vegetable ingredients on the structure and metabolic potential of the infant gut microbiome at a stage of development when solid foods are being introduced to their diet. The digested foods, presented to faecal bacteria at equivalent fibre concentrations, demonstrated different capacities to influence gut bacterial metabolism, as seen by changes in acid and gas metabolites, bacterial glucosidase enzyme activity and the bacterial composition.

The sugar analysis of the digested foods revealed that the foods vary in their monosaccharide composition. The higher proportion of glucose in the oats, pumpkin and sweetcorn digesta, potentially making more glucose available for bacterial metabolism, could be derived from resistant starch, and/or beta-glucans particularly for oats (given in Supplementary Table S1 online). These data are consistent with these digesta being higher in storage polysaccharides. For sweetcorn, some of the glucose could also be derived from xyloglucans, as indicated by the higher proportion of xylose, relative to the other starch-rich foods: oats, pumpkin and carrot. The digested carrot, blackcurrant, apple and both kiwifruit were lower in glucose and higher in cell wall sugars and uronic acids, reflecting the pectin-rich nature of these samples. Comparing only the pectin-rich foods further, blackcurrant had the lowest uronic acid/rhamnose molar ratio, and the lowest (arabinose + galactose)/rhamnose molar ratio, indicating that the cell walls are comprised mostly of a rhamnogalacturonan-I type pectin, with fewer side chains or branching, relative to the other foods. Carrot cell walls on the other hand, have higher proportions of arabinose and galactose, consistent with carrots being a dietary source of arabinogalactan^18^. The higher uronic acid/rhamnose ratio in gold-fleshed and green-fleshed kiwifruit suggests that the pectin is comprised more of homogalacturonan than of rhamnogalacturonan-I. The higher fucose content in apple suggests the presence of a hemicellulose, fucogalactoxyloglucan, while the higher mannose content in both gold-fleshed and green-fleshed kiwifruit suggests the presence of the hemicellulose, galactoglucomannan^19^. However, only the fruits (and not carrot) were high in xylose/glucose molar ratio, suggesting that the main hemicellulose present in the fruits is xyloglucan. The sugars thus available to the gut microbiota have previously been shown to be metabolised at different fermentation rates with diverse metabolic trajectories^20^. Using carbohydrate-free media supplemented with free sugars or sugar derivatives in faecal fermentations, glucose was shown to be fermented much more rapidly than free arabinose, rhamnose and xylose, or substrates with glucose and galactose substitutions or uronic acid derivatives. The acid metabolome produced was also distinct, e.g., glucose favoured lactate and acetate, while arabinose, xylose and fucose substitution increased propionate at the expense of lactate. We observed a similar propensity to increased propionate via propiogenic bacteria, with the highest propionate in apple, which had the highest fucose and xylose content. The slower fermentation is beneficial as it extends the availability of sugars towards the distal part of the colon, and mitigates the effects of a proteolytic fermentation to which the bacteria resort in the absence of sugar substrates^20^.

The inulin control, predominantly comprised of fructose (for which we did not assay) had the lowest concentrations of all other sugars, with those detected predominantly glucose and galactose and some uronic acid, consistent with the source and mode of extraction. We were unable to identify sugars in the digesta control; however, the sugar concentration may be inferred to be extremely low, and derived from the glycans or sugar-based extenders that are sometimes added to commercial digestive enzymes (pepsin, pancreatin and amylosidase)^21^. Fats and proteins, if present in the substrates, are presumed to be digested and dialysed out, although this was not confirmed by analyses.

The mean gastrointestinal transit time ranges from an average of 8.5 h for 1- to 2-month-olds, to 10 h for 1- to 2-year-olds^22^. These data, along with the high discrimination between the foods in terms of microbial glycosidases, gas and organic acid production, and microbiome changes, provide information on the potential prebiotic fermentability of the foods tested in this study. Given the high instability and inter-individual variability of infant gut microbiota, we created a more diverse inoculum by pooling faecal samples as an inoculum to assess the fermentability and prebiotic potential of foods with different carbohydrate assemblages^1,23^. We employed an anaerobic batch fermentation model to characterise the fermentability. The model was pH-controlled using buffering with carbonate/bicarbonate in the bacterial growth media, rather than through acid/base addition. Laboratory models simulating human digestion and colonic fermentation provide a rapid, well-validated means to examine bacterial changes in terms of ecology and metabolic capacity^24–26^. We did, however, observe a large increase in *Enterobacteriaceae;g__* which may be attributed to their much higher proportions in the infant inoculum and their flexible metabolic capacity enabling their growth in peptone-rich growth media^27^. These higher numbers of *Enterobacteriaceae;g__* may have had an unduly large influence on the gas and acid (mainly acetate) production.

Inulin was included as a standard prebiotic control, and it demonstrated the expected bifidogenic response, higher than for all the foods, thus validating our in vitro gut model. Such a bifidogenic response has been demonstrated in similar in vitro cultures with infant faecal bacteria and in clinical studies using inulin-supplemented infant formula^24,25^.

The effects of the foods on microbial metabolism were measured in terms of microbial gas and OA production. The microbial gas production was analysed in terms of gas pressure, rather than gas composition, and it is indicative of the complete catabolism of carbon sources to generate H_2_, CO_2_, and less often CH_4_, when methanogens are present^28^.

The production of gas and OA metabolites depends on the catabolic pathways employed by the bacteria to breakdown mono- and disaccharides. It is difficult to ascribe genus- or species-level identification using 16S rRNA gene sequencing data, with potential redundancies in functional and metabolic traits. However, interesting trends associating the microbiome with plant food carbohydrates emerge. While lactobacilli and bifidobacteria are not gas producers, clostridia employ rapid, heterofermentative saccharolytic or proteolytic pathways to generate acid and gas^28^. Thus although inulin is primarily bifidogenic, bifidobacteria-led increase in lactate (and acetate) may serve to feed clostridia, causing concurrent gas and butyrate production^28,29^. While *Bacteroides* are adept at metabolising glycans, they can also break down proteins in a carbohydrate-poor medium to generate gas^28,30^. The carbohydrate-poor digesta control thus showed increased gas, although its low total acid production may be attributed to the least amount of preferred or readily metabolisable substrate for gut microbial metabolism. This also explains the distinctive decrease in bifidobacteria. The increased populations of *Bacteroides* and clostridia in the water digesta thus potentially resort to their proteolytic capacity to survive, and thrive in the host digestive enzyme- and peptone-rich eutrophic environment^28,30^. Indeed, butyrate production is always associated with gas production^28,31^, but given the immaturity of the infant gut microbiome^1,2^, butyrate concentrations were much lower and increased to detectable amounts only in the inulin and digesta controls, and then only after 24 h. In concurrence, in a parallel experiment where the foods were inoculated with pooled adult faeces, we observed that the acid and gas formation increased much more at 5 h than it did with infant faeces and peaked at 16 h (data not presented).

The difference in composition of sugars and UA content, and the degree of branching, and other factors not measured in this study, such as diversity of types of sugar linkages, viscosity and degree of polymerisation may contribute to the fermentability of plant foods^12^. The absence of gas production with apple, blackcurrant, kiwifruit and pumpkin throughout the duration of the fermentation may be attributed to their fibre composition and the metabolic pathways employed by the bacteria. The pectin-rich polysaccharide proportions and the complexity of the pectin structure in terms of composition and linkages may lend the fruit substrates comparatively more resistance to fermentation, although this may increase the fermentable material in the distal colon, which is largely bereft of carbohydrates for the microbiota. In the case of apple, blackcurrant, gold-fleshed and green-fleshed kiwifruit, the polysaccharides had high xylose/glucose ratios. Xyloglucans require a complex glycanase machinery that is harboured by a limited number of *Bacteroides* and clostridia^13^. Thus, utilisation of xyloglycans is prioritised only after other low molecular weight or simpler glycans are metabolised. This explains the propiogenic effect and the absence of gas production from these fruit-based foods. This also explains the high proportionate increase in the RA of *Veillonella,* which are associated with a limited glycolytic activity, utilisation of lactate to generate propionate, and thus a tendency to increase when carbohydrates are only partially degraded^26^. There was also an increase in *Bacteroides* in the fruit fermenta. *Phascolarctobacterium* spp. are associated with acetate and propionate production, increasing in numbers with the host age, and their colonisation has been associated with improved mood^32^. Their inverse correlation with rhamnose content and direct correlation with uronic acid/rhamnose was most evident with the lowered *Phascolarctobacterium* in the rhamnose-rich blackcurrant, which also had the lowest uronic acid/rhamnose ratio.

Pumpkin, which also did not cause gas production, may have favoured a different bacterial metabolic pathway, indicated by its low propiogenic activity, wherein *Veillonella* was decreased, although *Bacteroides* was not suppressed. Pumpkin has a high glucose concentration (the highest molar% in this study) with a comparatively low xylose/glucose ratio, indicating an abundance of glucose that was easily available for microbial utilisation. This is consistent with the decreased *Veillonella* (lowest amongst the foods) and the increased *Bifidobacterium* (highest amongst the foods). The bifidobacteria adopt a homofermentative route for sugar metabolism, evidenced by the lactate and acetate concentrations (the highest in all the foods), which in turn caused the increase of cross-feeding clostridia such as *[Ruminococcus]*^33^. The highly lactogenic and acetogenic environment and the consequent lowered pH may explain the control of *Bacteroides* growth, and thereby the low propionate^34,35^. Pumpkin thus showed no gas production and had maximum *Bifidobacterium* and *[Ruminococcus]*.

Oats, sweetcorn and carrot increased lactate and acetate, and generated gas, consistent with the increased lactic acid bacteria, mainly bifidobacteria (and *Lactococcus* in case of carrots) that thrived on the high sugar content, including the β-glucan in the case of oats^26^. Also, these foods caused a comparative increase in the butyrogenic families of *Ruminococcaceae* and *Lachnospiraceae*, at the expense of *Bacteroides*. This overall microbiome profile may have contribute to the increased heterofermentative activity and the consequent gas production in these foods^28^. Inulin similarly drove increases in *Bifidobacterium*, *Bacteroides* and *Clostridium*, with concomitant gas production, indicative of increased heterofermentative activity^33^.

*Faecalibacterium* is the single most abundant genus from *Firmicutes* to colonise the adult gut and is associated with many benefits, including butyrate generation for colonocyte health^36^. Consistent with the correlation analyses, *Faecalibacterium* was increased in fucose-poor oats and sweetcorn and diminished in fucose-rich apple. Many *Bacteroidetes* members utilise their endo-xyloglucanase or employ the concerted action of α-xylosidases, β-galactosidases, and β-glucosidases to cleave sugars from the fucogalactoxyloglucans, such as those present in apples^37^.

Among the biggest needs of a growing infant are development of intestinal and immune maturity, both of which are associated with an increased microbial diversity, the biomarkers of which include selected clades of *Clostridiales*, notably from *Ruminococcaceae* and *Lachnospiraceae*^1,38,39^. The most stimulatory effect on well-known genera of these families was seen with pumpkin, oats, sweetcorn and carrot, rather than the fruits. Thus, the overall low xylose/glucose ratio in the oats and vegetables favours a beneficial shift that is of particular importance in the weaning infant’s gut, where the clostridia are initially low and increase only as new complementary foods are introduced from about 6 months of age^1,2^. While ultimately there are many factors that influences consumers’ preferences for infant complementary foods, this new knowledge on food-driven microbiome changes opens up opportunities to generate new concept food blends of different nutrient compositions, particularly carbohydrates, to drive microbiome diversity in the gut^3^.

Overall, the availability of glucose was a major microbiome modulator. It was seen to correlate with increases in *Bifidobacterium,* lactic acid bacteria and certain *Firmicutes* and concomitant suppression of *Bacteroidetes*. This may be explained by the constitutive expression of glucosidases for the utilisation of the most easily available energy source, before switching on costlier, energetically more expensive, polysaccharide utilisation pathways in *Bacteroides*^33,40^. There was a decrease in *Prevotella* but not *Bacteroides*, but this may have been an effect of the relative proportions of the two genera in the initial inoculum, rather than the measure of their glycosidase activity that can degrade both host and plant-glycans^41^. It may also be limited by the access of the bacteria to the sugars, especially in the pectin-rich apple and blackcurrant. Some of the microbial activity may also have been due to the polyphenolic content of the plant foods. Many polyphenols are poorly absorbed and up to 90% of these then concentrate in the large intestine to influence microbial activity^42–44^. Also, residual fat and protein-breakdown products from the substrates, if not broken down and dialysed out, may be carried over to the fermentation stage and therefore be available for bacterial metabolism. Indeed, it is possible that the digestive enzymes serve as carbon and nitrogen sources for gut bacteria^45^. However, we included a water digesta control that contained the same digestive enzymes, which thus allowed comparison across all the treatments. At 10 h of fermentation, all foods caused shifts in the microbial profile, leading to an abundance in bacteria that are capable of glycosidase activity and making available sugars for further microbial metabolism.

Clinical studies are ideal for evaluating the beneficial potential of prebiotic ingredients and foods, but such studies are time-consuming, expensive and fraught with ethical and logistic issues, especially when studying foods for infants and toddlers. For example, high drop-out rates due to like/dislike of foods are often observed. However, in vitro fermentations using validated models provide safe, high-throughput means of evaluation of prebiotic activity of promising ingredients for infant complementary foods^26^. While this study was conducted in a closed system, and thus cannot simulate acquisition of new microbes, it can reveal which microbes present in the newly weaned infant microbiome respond to different food carbohydrate composition, and suggest which niches may become further available for acquired microorganisms in the guts of infants consuming these foods.

In conclusion, this study shows that the monosaccharide composition of the plant-food fibre drives the infant gut’s metabolic and microbiome profile, at least under in vitro conditions. These in vitro studies provide a rapid and predictive option to determine food-microbiome interactions. Further in vitro studies would be useful to assess the impact of dosage and combination of ingredients on microbiome responses in the presence of milk, which is solely consumed in infants prior to or during the introduction of complementary foods.

## Materials and methods

### Infant complementary food ingredients

The foods included a cereal grain (oat flour) and commercially sourced purees from fruits (apple, de-seeded blackcurrant, green-fleshed and gold-fleshed kiwifruit) and vegetables (carrot, pumpkin and sweetcorn) (Table 1, and nutritional analysis data in Supplementary Table S1 online). The foods are referred to as oats, apple, blackcurrant, green-fleshed kiwifruit, gold-fleshed kiwifruit, carrot, pumpkin and sweetcorn hereafter.

The positive control was a commercial prebiotic, Orafti Synergy1 (hereafter referred to as inulin, ORAFTI Active Food Ingredients, Tienen, Belgium), a 1:1 mixture of short-chain oligofructans (degree of polymerisation < 10) and long-chain inulin (degree of polymerisation 2–60).

### Simulated gastric and ileal digestion

The foods were processed using previously published in vitro protocols simulating the digestion and absorption that occurs in the infant gut, as given in the workflow in Fig. 1. ^34,46^.The purees were used as given, while the oat and the positive control inulin were hydrated in sterile distilled water. Sterile water was included as a vehicle control. All the substrates (20 g of oat or 100 g of the puree) were digested by incubation at 37 °C with 0.03% w/v acidified pepsin, pH 2.5 for 30 min (gastric phase) followed by 0.18% w/v amyloglucosidase, 0.2% w/v pancreatin, pH 6.5 for 120 min (ileal phase). The final concentrations in the digesta in terms of fibre (using the composition data in Supplementary Table S1 online) were between 20 and 48 mg/mL for the different foods and 30 mg/mL for inulin. All the treatments were performed in triplicate. The digesta was then dialysed using 1000 Da molecular weight cut-off SpectraPor CE membrane (Thermo Fisher Scientific, Auckland, New Zealand) with at least four changes of cold deionised water, before being left overnight at room temperature.

The dialysed digesta was collected, a 3-mL aliquot freeze-dried for sugar composition analysis and the remaining digesta was used for anaerobic fermentation with infant faecal bacteria.

### Analysis of fibre-derived sugar composition

Neutral sugar composition of the digested foods was determined as described previously^47^. Three-mL digesta from each substrate was freeze-dried and 1–2 mg hydrolysed in 2 M trifluoroacetic acid for non-cellulosic sugars, derivatised into alditol acetates, and quantified by gas chromatography with a flame ionisation detector, using inositol as internal standard and in reference to the sugar standards rhamnose, fucose, arabinose, xylose, mannose, galactose and glucose. Samples were measured in duplicate, each with duplicate injections.

Uronic acid composition of foods was also determined by the m-hydroxydiphenyl method^47^. The freeze-dried samples (1–2 mg) were hydrolysed in concentrated H_2_SO_4_, and a 1:5 diluted sample (0.04 mL) assayed in 96-well, optically clear, flat-bottomed microplates with H_2_SO_4_ containing 75 mM sodium tetraborate reagent (0.2 mL) for 1 h at 90 °C, and the colour reaction was carried out by adding dinitrophenyl colour reagent (0.01 mL). Absorbance was read at 520 nm against galacturonic acid standards.

### Collection of faecal samples

Fresh faecal samples were obtained from five healthy infants (between the ages of 6 and 10 months), with written consent, approval #16/NTA/151 from Health and Disability Ethics Committees, Ministry of Health, New Zealand. The infants had never taken any antibiotics or medications.

Immediately after voiding, nappy liners containing the faecal samples were transferred to anaerobic conditions in gas-tight bags with one Anaeropouch (Mitsubishi Gas Chemical Company, Inc., Tokyo, Japan), and chilled with ice packs while transported to the laboratory. The samples were processed within 1 h into 20% v/v faecal slurries in chilled, sterile, anaerobic, glycerol-buffered saline with 0.05% w/v cysteine, aliquots dispensed and stored at − 80 °C. All processing of faecal samples and subsequent inoculation for the fermentation was carried out in a controlled atmosphere of 5% CO_2_, 5% H_2_ and 90% N_2_ within the anaerobic chamber (Coy Laboratory Products Inc., Michigan, USA).

### Simulated colonic fermentation and headspace gas measurement in fermenta

Fermentations were conducted in Hungate tubes under CO_2_, sealed with butyl rubber septa. The food digesta were standardised to a final fibre concentration of 3 mg/mL of fermentation volume using the fibre concentrations given in Supplementary Table S1 online. Inulin was similarly fermented at a final concentration of 3 mg/mL. Water digesta was included as a digesta control, to evaluate the potential effects of the digestion enzymes that may remain after the dialysis step. Just before the fermentation, one aliquot of frozen faecal slurry from each donor was thawed in the anaerobic chamber and pooled in equal proportions. All fermentations were carried out in triplicate, and inoculated with the pooled faeces at 1% w/v in a sterile, pre-reduced, carbohydrate-free basal medium, prepared using previously detailed proportions^34^. Two 1-mL aliquots were collected from the fermenta at 0, 5, 10, 16 and 24 h, immediately centrifuged at 13,000×*g* for 5 min at 4 °C, and the pellets and supernatants stored at − 80 °C. The pellets were used for extraction of DNA, while the supernatants were used for assay of microbial glycosidases and OAs. Immediately prior to sampling, fermentation headspace gas pressures were measured by penetrating the rubber septum with a 22-ga needle attached via Luer connection to a pressure gauge (WIKA EN 837-1 14.5 PSI/100 kPa max, WIKA Instruments Ltd, Auckland, New Zealand).

### Analysis of organic acids

Fermenta supernatants from all collection time points were analysed for the OAs, formate, acetate, propionate, isobutyrate, isovalerate, valerate, hexanoate, lactate and succinate using gas chromatography^21^. The supernatants (0.1 mL) were diluted in 4 volumes of phosphate-buffered saline containing the internal standard 2-ethylbutyrate to a final concentration of 5 mM, protonated in concentrated HCl (0.25 mL), and partitioned into diethyl ether (1.0 mL). This organic extract was derivatised with N-tert-butyldimethylsilyl-N-methyltrifluoroacetamide with 1% tert-butyldimethylchlorosilane at 80°C for 20 min, then room temperature for 48 h to allow complete derivatisation. Analysis was performed on a flame ionisation detector-equipped capillary gas chromatography system (GC-2010 Plus; Shimadzu, Kyoto, Japan) fitted with a Restek column (Rtx-1, 30 m × 0.25 mm × 0.25 µm) (Bellefonte, PA, USA). The carrier gas was helium with a total flow rate of 21.2 mL/min and pressure of 131.2 kPa. Make-up gas was nitrogen. The temperature program began at 70 °C, increasing to 115 °C at 6 °C/min, with a final increase to 300 °C at 60 °C/min, holding for 3 min. Flow control mode was set to linear velocity of 37.5 cm/s. The injector temperature was 260 °C and detector temperature was 310 °C. Samples were injected (1 µL) with a split injection (split ratio: 10:1). The instrument was controlled, and data processed using Shimadzu GC Work Station LabSolutions Version 5.3 software. The OAs were quantified with reference to a mix of the authentic standards and presented as µmol organic acid per mL fermenta.

### Microbial glycosidase activity

Supernatants from the 0- and 10-h fermenta were analysed for their glycosidase activity through their ability to degrade para-nitrophenyl-1-linked sugars. Sugars and linkages that were represented included α-fucopyranose, α- and β-glucopyranose; α- and β-galactopyranose, α-arabinopyranose and α-arabinofuranose; α-N-acetylgalactosaminide; β-N-acetylglucosaminide; α-rhamnopyranose, α-mannopyranose and β-xylopyranose. Supernatants (0.005 mL) were pipetted into 384-well, optically clear microplates preloaded with 10 mM Tris.Cl buffer pH 7.4 (0.01 mL) and corresponding 4-nitrophenyl-labelled sugar derivatives (0.005 mL). Release of the 4-nitrophenol chromophore occurs after hydrolysis of the glycosidic linkage to the sugar. After 90 min of incubation at 37 °C, the reaction was terminated and liberated 4-nitrophenol colour developed by the addition of 0.5 M sodium glycine buffer pH 9.6 (0.05 mL), and absorbance was measured at 405 nm wavelength using a SPECTRAmax plus microplate reader (Molecular Devices Pty Ltd, Surrey Hills, VIC). All samples and standards were prepared in triplicate. Given the large differences in activity with different substrates, as key activities are in much greater abundance and activity in faecal microbiota (e.g. galactosidases, glucosidases, N-acetylglycosaminidases) relative to much lower plant-glycan specific activities (e.g., rhamnosidases and xylosidases), we elected to transform the enzyme activities into percentages relative to the starting (0 h) fermenta, to better illustrate changes in response to treatments.

### Extraction of DNA from fermenta

DNA was extracted from the pellets of the fermenta obtained at all time points (0, 5 and 10 h) using the PowerSoil DNA Isolation kit (MO Bio Laboratories, California, USA), according to the manufacturer’s instructions, with the following modifications. The pellets were dispersed in the PowerBead tubes, to which 60 µL of solution C1 was added. This was followed by mechanical shaking using FastPrep-24 5G (MP Biomedicals, Ohio, USA), with 4 cycles of 5.5 m/s for 60 s, with the tubes being kept in ice for 5 min between each cycle. The tubes were then frozen at -80 °C and all further steps carried out as per the manufacturer’s instructions within 48 h.

The quantity and quality of extracted DNA were estimated using the QIAxpert System (Qiagen sourced from Bio-Strategy Ltd, Auckland, New Zealand).

### Microbiome characterisation using 16S rRNA gene sequencing

The DNA from the 5- and 10-h fermenta, along with the 0-h sample of the water and inulin control, were submitted to Massey Genome Service (Palmerston North, New Zealand) for preparation of amplicon libraries for the 16S rRNA gene V3-V4 regions using the previously described 341F and the 806R primers, with custom barcodes for downstream sequencing^48^. The purified amplicons were then pooled in equal molarity and the sequencing performed using 2 × 250 base paired-end runs using the Illumina MiSeq v. 2.0 platform^48^.

### Bioinformatics

Quantitative Insights Into Microbial Ecology (QIIME) software version 1.9.1 was used to analyse the sequencing data^49^. To assemble the paired-end reads into a single continuous sequence, PANDASeq was used with parameters of at least 40 bp overlap, a minimum of 350 bp length and maximum of 500 bp^50^. Putative chimeras were filtered from the sequences and the reads clustered into operational taxonomic units (OTUs) based on a 97% identity threshold value using USEARCH and UCLUST^51^. Alignment of the sequences was carried out using PyNAST^52^ with reference to the Greengenes core reference database (version 13_8)^53^. Microbial α- and β-diversity analyses were performed using 35,000 reads, and visualised as barplots and 2-D principal co-ordinate analysis plots respectively^54^.

### Statistical analyses

For analysis of sugar composition, microbial acid and gas production, enzyme activity and microbiome analysis, data were analysed at individual time points. Single-factor analysis of variance was used, with substrate (i.e., foods, inulin or water digesta) as the factor. Usually data were log-transformed to stabilise the variance. For the microbiome counts, differential abundance analysis, using DESeq2^55^ with likelihood ratio tests and a nested factorial structure, allowed testing for differences between the treatments at each time point, and then between the averages for each time-point. The *p* values were adjusted for false discovery rate. Multiple comparisons between substrate means were made using Tukey’s Honestly Significant Difference (HSD) at the 5% level.

The changes in the OA were visualised as a Principal Components Analysis (PCA) plot using MetaboAnalyst 4.0, www.metaboanalyst.ca^56^.

Microbial diversity within samples was measured using the Shannon index and the significance calculated using the Kruskal–Wallis test. The diversity between samples was calculated using the Bray–Curtis distances between samples, and the significance calculated using Analysis of group Similarities with MicrobiomeAnalyst, www.microbiomeanalyst.ca^54^.

Spearman rank correlation test was used to analyse correlations between fibre-derived sugars as concentrations and molar %, the 10-h fermenta microbial glucosidase activity and the 10-h fermenta microbiome. Significant correlation was examined and given as a false discovery rate-adjusted (FDR) *P* value.

### Ethical approval

All methods were carried out in accordance with relevant guidelines and regulations of the Health and Disability Ethics Committees, Ministry of Health, New Zealand. Informed consent was obtained from a parent and/or legal guardian, as infants were involved in the study.

## Supplementary Information

Supplementary Information

## Data Availability

The metadata and the 16S rRNA gene sequence data generated during this study have been deposited with links to BioProject accession number PRJNA663722 in the NCBI BioProject database (https://www.ncbi.nlm.nih.goc/bioproject/).
