# Supplementary material for: The sugar composition of the fibre in selected plant foods modulates weaning infants’ gut microbiome composition and fermentation metabolites in vitro
Source: Sci Rep. 2021 Apr 29;11:9292. doi: 10.1038/s41598-021-88445-8 (PMC8085221; doi:10.1038/s41598-021-88445-8)
Supplement: Supplementary file 1 — Supplementary Information [file 41598_2021_88445_MOESM1_ESM.pdf]

Supplementary Material

The sugar composition of the fibre in selected plant foods modulates weaning infants' gut microbiome composition and fermentation metabolites *in vitro*.

Shanthi G. Parkar*[1], Jovyn K.T. Frost[1], Doug Rosendale[1], Halina M. Stoklosinski[1], Carel M. H. Jobsis[1], Duncan I. Hedderley[1], Pramod Gopal*[1,2].

[1]The New Zealand Institute for Plant and Food Research Limited, Private Bag 11600, Palmerston North 4442, New Zealand.

[2]Riddet Institute, Massey University, Palmerston North 4442, New Zealand.

*Correspondence: S.G.P. shanthi.parkar@gmail.com ; P.G. Pramod.Gopal@plantandfood.co.nz

Table S1. Nutrient compositional analysis of the infant complementary foods included in this study.

**Ingredient**		**Oats**	**Blackcurrant**	**Carrot**	**Apple**	**Green-fleshed kiwi fruit**	**Gold-fleshed kiwi fruit**	**Pumpkin**	**Sweetcorn**
Product name		Oat flour	Blackcurrant puree, seedless	Carrot puree, aseptic	Apple puree	Green kiwifruit puree, seed-out	Gold kiwifruit puree, seed-out	Pumpkin puree, frozen	Sweetcorn puree, frozen
Product code		F00005	BLC2001	CAR5100	Royal Gala	SP-01-016	SP-01-005	Frozen squash puree	Frozen corn puree
Supplier		Harraways	JP-NZ	JP-NZ	Frupak	Kiwifruitz	Kiwifruitz	Cedenco	Cedenco
Quantity		100 g	100 g	100 g	100 g	100 g	100 g	100 g	100 g
	Unit								
Energy	kJ	1468	184	167	143	186	216	249	292
Total nitrogen	% m/m	2.254	0.079	0.124	0.022	0.087	0.119	0.184	0.359
Protein	g	13.14	0.49	0.78	0.14	0.54	0.74	1.15	2.24
Fat - total	g	6.5	< 0.1	0.2	< 0.1	< 0.1	0.1	0.1	1.4
Fat - saturated	g	1.2	< 0.1	< 0.1	< 0.1	< 0.1	< 0.1	< 0.1	0.3

Fat - mono-unsaturated	g	2.6	< 0.1	< 0.1	< 0.1	< 0.1	< 0.1	< 0.1	0.6
Fat - polyunsaturated	g	2.7	< 0.1	0.1	< 0.1	< 0.1	< 0.1	< 0.1	0.6
Sugar - total	g	0.8	9.3	7.6	7.6	9.6	11.4	5.7	6.4
Sugar - glucose	% m/m	< 0.1	1.6	< 0.5	3.4	4.6	5.4	1.9	< 0.5
Sugar - fructose	% m/m	< 0.1	6.3	< 0.5	4.2	5	6	1.7	< 0.5
Sugar - maltose	% m/m	< 0.1	< 0.1	< 0.1	< 0.1	< 0.1	< 0.1	< 0.1	< 0.1
Sugar - lactose	% m/m	< 0.1	< 0.1	< 0.1	< 0.1	< 0.1	< 0.1	< 0.1	< 0.1
Sugar - sucrose	% m/m	0.8	1.4	7.1	< 0.1	< 0.1	< 0.1	2.1	6.1
Starch	g	54.3	< 0.1	0.2	< 0.1	0.2	0.1	6.6	4.4
Fibre - total	g	8.4	2.2	1.7	1.5	1.3	1.0	2.6	2.3
Fibre - insoluble	g	5.4	1.3	0.8	1.0	0.7	0.7	2.1	2.0
Fibre - soluble	g	3.1	0.9	0.8	0.4	0.5	0.3	0.5	0.3
Fibre - beta-glucan	g	3.3	-	-	-	-	-	-	-
Sodium	mg	2.6	3.1	54	2.5	3.1	2.6	0.45	0.48
Potassium	mg	340	230	400	100	310	300	320	240
Calcium	mg	61	21	29	4.7	19	12	12	2
Magnesium	mg	120	8.6	16	4	8.7	9.3	13	23

Iron	mg	4	0.76	0.87	0.098	0.16	0.23	0.44	0.4
Phosphorous	mg	380	18	42	8	15	16	23	68
Vitamin A	µg RE*	0.363	8.95	1783	0.38	3.9	4.7	228.72	4.36
Thiamin (Vitamin B1)	mg	0.63	0.02	0.04	0.01	< 0.01	0.01	0.05	0.07
Riboflavin (Vitamin B2)	mg	0.08	0.03	0.06	0.07	0.08	0.07	0.08	0.08
Niacin (Vitamin B3)	mg	0.52	0.73	0.56	< 0.01	0.12	0.17	0.94	1.655
Vitamin C	mg	< 1	125.3	< 1	< 1	63.25	82.24	< 1	< 1
Moisture	% m/m	12.96	84.0	87.5	86.8	84.1	82.5	80.5	81.3
Ash	% m/m	1.7	0.4	0.8	0.2	0.4	0.4	0.6	0.5

*RE = retinol equivalent.

Table S2. Molar percentages and ratios of neutral sugar and uronic acid composition of the digested foods. Values (mol %) are mean values or ratios calculated from absolute concentrations in Table 2 of the main paper. Rha = rhamnose, fuc = fucose, Ara = arabinose, Xyl = xylose, Man = mannose, Gal = galactose, Glc = glucose, UA = uronic acid, KF = kiwifruit.

**Sugar**	**Apple**	**Blackcurrant**	**Carrot**	**Green-fleshed kiwifruit**	**Gold-fleshed kiwifruit**	**Inulin**	**Oats**	**Pumpkin**	**Sweetcorn**
Molar percentage									
Rhamnose	2.5	6.2	3.5	1.8	1.8	0.6	0.1	1.4	0.6
Fucose	1.4	0.5	0.3	0.6	0.5	0.8	0.0	0.2	0.1
Arabinose	9.4	5.7	11.2	3.3	3.1	3.9	2.9	3.3	6.6
Xylose	5.8	5.7	1.4	5.9	5.6	0.6	2.4	1.6	5.1
Mannose	1.9	1.9	2.2	2.2	2.7	4.6	0.7	1.5	0.8
Galactose	7.3	6.9	16.5	9.7	9.5	16.0	2.4	9.3	5.4
Glucose	34.8	36.4	30.0	41.1	44.3	58.0	86.8	65.6	68.8
UA	36.9	36.6	34.9	35.4	32.5	15.5	4.7	17.1	12.5
Molar ratio									
UA/Rha	15.0	5.9	9.1	19.7	18.3	24.2	42.4	11.9	22.0
Gal + Ara/Rha	6.8	2.0	2.0	7.2	7.2	31.1	48.4	8.8	21.1
Xylose/glucose	0.2	0.2	0.0	0.1	0.1	0.0	0.0	0.0	0.1

Table S3. Limits of detection (LoD) of the organic acids, measured as µmol organic acid/mL fermenta

	Formate	Acetate	Propionate	Isobutyrate	Butyrate	Isovalerate	Valerate	Hexanoate	Heptanoate	Lactate	Succinate
LoD	0.3	1	0.42	0.3	1	0.1	0.1	0.1	0.1	0.25	0.3

Table S4. Organic acid concentrations at each time point after fermentation. Values are average organic acid concentrations in µmol/mL of fermenta (n=3). The average Coefficient of Variability (CV) expressed as a percentage is also presented. The significance value * P = 0.028; all other P values for each acid in terms of ingredient effect were < 0.001.

Group	Apple	Blackcurrant	Carrot	Digesta control	Gold-fleshed kiwi fruit	Green-fleshed kiwi fruit	Inulin	Oats	Pumpkin	Sweetcorn	Average CV
5 h											
Formate	1.2^{c}	0.6^{a}	1.2^{c}	2.2^{d}	1.0^{bc}	0.9^{b}	2.1^{d}	1.1^{bc}	0.9^{b}	1.2^{c}	8.5
Lactate	1.5^{b}	1.2^{b}	3.5^{cd}	0.3^{a}	3.2^{cd}	3.3^{cd}	0.9^{bc}	5.4^{de}	6.6^{e}	4.9^{de}	27.3
Acetate	7.0^{c}	4.8^{a}	7.3^{cd}	5.6^{b}	8.0^{d}	8.0^{d}	6.4^{cd}	9.8^{e}	10.3^{e}	9.5^{e}	4.1
Propionate	1.2^{de}	0.8^{a}	1.1^{bc}	1.4^{f}	1.1^{b}	1.2^{bcd}	1.1^{b}	1.2^{cde}	1.2^{bcd}	1.3^{ef}	4.3
10 h											
Formate	0.8^{d}	0.5^{bc}	0.7^{cd}	2.8^{e}	0.4^{ab}	0.5^{bc}	1.6^{cd}	0.3^{a}	0.4^{ab}	0.5^{bc}	14.3
Lactate	2.1^{b}	0.3^{a}	5.1^{c}	0.3^{a}	4.1^{bc}	3.8^{bc}	2.3^{c}	5.0^{bc}	8.7^{c}	5.2^{c}	31.1
Acetate	11.9^{c}	9.6^{b}	12.7^{cd}	7.4^{a}	12.0^{c}	12.3^{cd}	9.7^{d}	11.9^{c}	13.8^{cd}	12.7^{cd}	7.8
Propionate	3.2^{e}	2.4^{cd}	2.3^{bcd}	2.0^{abc}	2.4^{cd}	2.6^{de}	1.9^{ab}	1.7^{a}	1.6^{a}	2.4^{cd}	5.3
16 h											
Formate	0.4^{ab}	0.4^{b}	0.3^{a}	1.8^{c}	0.3^{a}	0.3^{a}	1.0^{ab}	0.3^{a}	0.3^{a}	0.3^{a}	10.5
Lactate	0.4^{a}	0.3^{a}	2.5^{b}	0.3^{a}	2.5^{b}	0.7^{a}	2.5^{bc}	5.9^{bc}	9.0^{c}	0.5^{a}	43.5
Acetate	13.9^{c}	12.5^{b}	13.3^{bc}	5.9^{a}	14.1^{c}	14.5^{c}	10.2^{d}	13.9^{c}	14.5^{c}	13.5^{bc}	7.9
Propionate*	4.3^{b}	3.4^{ab}	3.1^{ab}	2.2^{ab}	2.4^{ab}	4.0^{b}	2.1^{ab}	2.2^{ab}	1.3^{a}	3.7^{ab}	16.2
24 h											
Formate	0.3^{a}	0.3^{a}	0.3^{a}	0.9^{c}	0.3^{a}	0.3^{a}	0.6^{b}	0.3^{a}	0.3^{a}	0.3^{a}	4.2

Lactate	0.3^{a}	0.3^{a}	0.3^{a}	0.3^{a}	0.6^{a}	0.3^{a}	2.7^{b}	7.9^{b}	10.1^{b}	0.3^{a}	28.8
Acetate	14.8^{bcd}	14.0^{b}	14.4^{bc}	5.1^{a}	15.2^{bcd}	15.7^{d}	10.2^{e}	15.5^{cd}	15.7^{d}	14.0^{b}	8.4
Propionate	4.9^{f}	3.7^{d}	3.9^{d}	2.3^{b}	4.3^{e}	4.8^{f}	2.2^{b}	2.6^{c}	1.8^{a}	4.6^{ef}	2.8
Butyrate	1.0^{a}	1.0^{a}	1.0^{a}	1.1^{b}	1.0^{a}	1.0^{a}	1.1^{a}	1.0^{a}	1.0^{a}	1.0^{a}	0.3

Table S5. Change in glycosidase activities against colorimetric substrates by faecal microbiota fermented with substrates for 10 h. Data mean of three replicates and expressed as percentage of activity at 0 h, with average standard error of mean. The enzymes included α-arabinofuranosidase (α-araF), α-arabinopyranosidase(α-araP), α-fucopyranosidase (α-fucP), α- and β-glucopyranosidase (α-glcP, β-glcP), β-glucopyranosidase α- and β-galactopyranosidase (α-galP, β-galP), and α-N-acetylgalactosaminidase (α-galNAc); β-N-acetylglucosaminidase (β-glcNAc); α-rhamnopyranosidase (α-rhaP), α-mannopyranosidase (α-manP) and β-xylopyranosidase (β-xylP). The significance values are adjusted to the false discovery rate.

Substrate	Apple	Blackcurrant	Carrot	Digesta control	Gold-fleshed kiwifruit	Green-fleshed kiwifruit	Inulin	Oats	Pumpkin	Sweetcorn	SEM	*P* value
α-AraF	57^{ab}	57^{ab}	49^{a}	79^{b}	59^{ab}	49^{a}	47^{a}	54^{a}	59^{ab}	54^{a}	5	0.01
α-AraP	121^{ab}	103^{ab}	120^{ab}	97^{a}	129^{b}	109^{ab}	110^{ab}	101^{ab}	100^{ab}	110^{ab}	6	0.02
α-FucP	107^{a}	88^{a}	104^{a}	89^{a}	103^{a}	91^{a}	98^{a}	91^{a}	87^{a}	93^{a}	5	0.04
α-GalP	72^{abc}	53^{a}	60^{ab}	99^{bc}	63^{ab}	55^{a}	106^{c}	59^{ab}	78^{abc}	66^{abc}	8	0.002
α-GalNAc	132^{a}	97^{a}	104^{a}	102^{a}	113^{a}	109^{a}	86^{a}	121^{a}	108^{a}	96^{a}	12	0.32
α-GlcP	76^{ab}	67^{a}	73^{a}	95^{b}	74^{a}	67^{a}	135^{c}	67^{a}	78^{ab}	70^{a}	4	< 0.001
α-ManP	101^{a}	88^{a}	114^{a}	89^{a}	131^{a}	124^{a}	101^{a}	119^{a}	111^{a}	112^{a}	19	0.82
α-RhaP	139^{a}	122^{a}	108^{a}	100^{a}	122^{a}	114^{a}	76^{a}	128^{a}	105^{a}	93^{a}	16	0.27
β-GalP	101^{ab}	92^{ab}	105^{ab}	88^{a}	101^{ab}	96^{ab}	115^{b}	88^{a}	89^{a}	102^{ab}	5	0.01
β-GlcP	101^{ab}	83^{a}	92^{a}	91^{a}	101^{ab}	91^{a}	128^{b}	96^{ab}	94^{ab}	96^{ab}	7	0.02
β-GlcNAc	82^{bc}	59^{ab}	76^{abc}	98^{c}	74^{abc}	66^{ab}	79^{abc}	52^{a}	64^{ab}	73^{abc}	6	0.001
β-XylP	121^{a}	110^{a}	105^{a}	92^{a}	114^{a}	104^{a}	89^{a}	112^{a}	102^{a}	93^{a}	11	0.54

Table S6. The average relative abundance of the bacterial phyla in the 0-h fermentation sample.

Phylum	Relative Abundance	
	Average	Standard Deviation
Actinobacteria	21.385	4.073
Bacteroidetes	28.951	5.810
Proteobacteria	9.594	1.134
[Thermi]	0.000	0.001
Bacteria;Other	0.000	0.000
Unassigned;Other	1.513	0.323
Chloroflexi	0.000	0.000
Cyanobacteria	0.001	0.001
Firmicutes	38.499	1.119
Fusobacteria	0.010	0.001
Planctomycetes	0.000	0.000
Spirochaetes	0.000	0.000
TM7	0.002	0.001
Tenericutes	0.000	0.000
Verrucomicrobia	0.045	0.033
WPS-2	0.000	0.000

Table S7. The average relative abundance of the bacterial genera in the 0-h fermentation sample. A total of 250 genera or groups at level 6 taxonomic level were identified. Genera with values greater than 0.05% relative abundance in at least one sample were included in the Table above.

Genus	Relative Abundance	
	Average	Standard Deviation
Bifidobacterium	16.81	3.03
Collinsella	4.16	0.93
Bacteroides	19.57	6.52
Prevotella	8.11	0.96
Lactococcus	0.01	0.02
Clostridium	0.30	0.14
[Ruminococcus]	7.89	0.97
Ruminococcaceae;g__	5.59	2.32
Phascolarctobacterium	2.05	1.04
Veillonella	5.15	1.25
Enterobacteriaceae;g__	8.13	0.81

Table S8. Average counts of taxonomic groups at family level that were significantly changed at the end of 5 h of fermentation. Values are average counts (n=3). The average Coefficient of Variability (CV) expressed as a percentage is presented. The significance values adjusted to the false discovery rate were * $P < 0.05$, ** $P < 0.005$, the likelihood ratio test adjusted for 32 tests. The inulin control values are not given as the sequence quality was poor and the data were disregarded.

Group	Apple	Black currant	Carrot	Digesta control	Gold-fleshed kiwifruit	Green-fleshed kiwifruit	Oats	Pumpkin	Sweetcorn	Average CV
*Not_Assigned***	2,218[a]	826[b]	1,231[a]	1,581[a]	1,318[a]	1,229[ab]	1,297[a]	1,271[ab]	1,484[a]	35%
*Bifidobacterium***	22,824[a]	17,056[a]	12,611[a]	9,538[b]	13,870[a]	18,419[a]	14,801[a]	13,592[ab]	15,606[a]	31%
*Collinsella**	2,091[ab]	1,933[a]	1,653[ab]	1,535[b]	1,510[ab]	1,814[ab]	1,508[ab]	1,607[ab]	1,853[ab]	31%
*Eggerthella***	394[ab]	183[a]	259[b]	363[b]	210[ab]	273[ab]	248[ab]	242[ab]	296[ab]	37%
*Bacteroides***	6,436[ab]	4,985[ab]	3,414[ac]	5,755[ab]	5,191[ab]	6,054[b]	3,248[c]	5,135[ab]	1,727[d]	34%
*Parabacteroides***	513[ab]	368[ab]	284[ac]	550[b]	433[ab]	512[ab]	219[cd]	447[ab]	162[d]	37%
*Prevotella***	5,067[a]	595[b]	4,152[a]	5,365[a]	3,201[a]	4,256[a]	5,286[a]	4,632[a]	4,839[a]	27%
*Enterococcus***	992[ab]	175[c]	515[a]	1,178[d]	596[a]	699[a]	1,159[d]	883[b]	1,517[e]	31%
*Lactobacillus***	166[abc]	66[a]	84[ab]	109[ab]	127[bc]	120[abc]	135[bc]	156[bc]	197[c]	31%
*Lactococcus***	1.33[a]	2.67[a]	4,501.67[b]	3.67[a]	3.33[a]	3.33[a]	4.33[a]	2.00[a]	4.33[a]	63%
*Streptococcus***	1,458[ab]	1,050[a]	762[c]	988[bc]	1,073[a]	1,108[abc]	983[abc]	989[abc]	1,133[ab]	33%
*Clostridiaceae;other***	884[ab]	852[c]	578[a]	545[b]	648[a]	762[a]	577[ab]	596[ab]	648[ab]	34%
*Clostridiaceae**	382[ab]	317[a]	241[ab]	249[b]	283[ab]	329[ab]	236[b]	275[ab]	292[ab]	33%
*Clostridium***	439[a]	40[b]	286[a]	1,297[c]	158[de]	147[d]	297[a]	188[e]	301[a]	31%

*Blautia***	177^{a}	191^{b}	135^{ab}	120^{a}	121^{a}	119^{a}	126^{a}	156^{ab}	117^{a}	35%
*Coprococcus***	268^{ab}	217^{ac}	149^{bd}	632^{e}	255^{c}	399f	137^{d}	62g	91h	33%
*Dorea***	1,599^{ab}	541^{c}	899^{ad}	2,378^{e}	650^{c}f	766f	1,239^{b}	1,136^{ab}	888^{d}	33%
[Ruminococcus]**	5,108^{ab}	2,238^{a}	2,518^{ab}	2,888^{ab}	2,955^{ab}	3,772^{ab}	3,944^{b}	3,613^{ab}	3,639^{ab}	28%
*Ruminococcaceae***	486^{abc}	382^{ab}	385^{a}	183^{d}	293^{bc}	310^{c}	327^{abc}	373^{abc}	391^{abc}	37%
*Faecalibacterium***	79^{a}	69^{ab}	85^{b}	39^{c}	61^{a}	57^{ac}	73^{ab}	76^{ab}	113^{b}	35%
*Oscillospira***	340^{ab}	153^{c}	208^{ab}	331^{a}	204^{bc}	251^{ab}	234^{ab}	243^{ab}	229^{bc}	34%
*Dialister***	824^{ab}	688^{c}	538^{ac}	481^{b}	626^{ac}	704^{ac}	583^{ac}	644^{ac}	650^{ac}	33%
*Phascolarctobacterium**	5,007^{ab}	2,602^{a}	2,962^{ab}	4,299^{b}	3,230^{ab}	3,863^{ab}	3,101^{ab}	3,020^{a}	3,432^{ab}	35%
*Veillonella***	11,178^{abc}	8,044^{a}	6,378^{abc}	6,082^{abc}	7,630^{ab}	9,323^{a}	5,341^{bc}	5,281^{c}	6,733^{abc}	35%
*Erysipelotrichaceae;other***	0.33^{ab}	0.00^{c}	0.00^{bc}	0.00^{a}	0.00^{bc}	0.00^{c}	0.00^{a}	0.00^{ab}	0.00^{a}	173%
*Sutterella***	181^{a}	189^{b}	139^{ab}	80^{c}	159^{ab}	211^{b}	154^{ab}	152^{ab}	127^{a}	37%
*Enterobacteriaceae;g__**	17,361^{ab}	11,739^{a}	9,109^{ab}	13,712^{a}	10,001^{ab}	12,239^{ab}	12,050^{ab}	9,566^{b}	10,752^{ab}	29%

Table S9. Average counts of taxonomic groups at genus level that were significantly changed at the end of 10 h of fermentation. Values are average of 3 counts. The average Coefficient of Variability (CV) expressed as a percentage is presented. All the P values were < 0.005, with the likelihood ratio test, adjusted for 32 tests. Pairwise differences within each bacteria, adjusted for FDR among 36 pairwise comparisons.

Group	Apple	Black currant	Carrot	Digesta control	Gold-fleshed kiwifruit	Green-fleshed kiwifruit	Inulin	Oats	Pumpkin	Sweetcom	Average CV
Not_Assigned	4,151^{a}	2,570^{ab}	3,522^{a}	4,562^{a}	3,048^{ab}	3,148^{ab}	2,990^{a}	3,339^{ab}	2,326^{b}	4,162^{ab}	23%
Bifidobacterium	36,649^{a}	43,968^{ab}	40,331^{ab}	19,439^{c}	45,973^{ab}	48,569^{ab}	28,289^{ab}	49,060^{ab}	56,750^{b}	57,693^{ab}	16%
Collinsella	3,580^{a}	3,420^{a}	3,977^{a}	2,728^{b}	4,292^{a}	4,187^{a}	1,635^{b}	4,390^{a}	4,059^{a}	5,448^{a}	19%
Eggerthella	937^{a}	431^{b}	770^{a}	1,588^{c}	759^{a}	761^{a}	403^{b}	1,068^{a}	867^{a}	1,196^{a}	20%
Bacteroides	11,239^{ab}	10,787^{abc}	8,612^{a}	12,188^{bc}	11,611^{abc}	15,506^{c}	19,588^{d}	7,413^{e}	11,950^{ab}	1,637^{f}	19%
Parabacteroides	678^{ab}	622^{ab}	551^{ab}	984^{a}	709^{a}	978^{a}	423^{ab}	472^{b}	651^{ab}	181^{c}	25%
Prevotella	967^{abc}	374^{ad}	1,477^{bce}	11,464^{f}	1,685^{be}	3,717^{eg}	4,868^{fg}	439^{ad}	649^{acd}	332^{d}	31%
Enterococcus	3,263^{ab}	508^{c}	2,386^{ad}	2,140^{d}	2,191^{d}	2,095^{d}	2,859^{be}	3,919^{ab}	2,766^{ad}	7,449^{e}	25%
Lactobacillus	721^{a}	244^{b}	451^{cd}	166^{e}	794^{a}	632^{ac}	288^{d}	1,647^{f}	1,382^{f}	3,268^{g}	16%
Lactococcus	3.67^{ab}	1.67^{a}	11,457.62^{c}	4.00^{ab}	5.33^{ab}	5.67^{ab}	8.33^{b}	10.33^{b}	4.00^{ab}	9.33^{ab}	48%
Streptococcus	2,051^{ab}	1,617^{abc}	2,042^{a}	1,496^{c}	2,352^{a}	2,034^{abc}	1,083^{bc}	2,624^{a}	2,534^{a}	2,853^{a}	18%
Clostridiaceae;other	1,278^{a}	1,340^{ab}	1,483^{ab}	840^{c}	1,528^{ab}	1,575^{ab}	568^{c}	1,809^{ab}	1,883^{b}	2,006^{ab}	19%
Clostridiaceae	831^{a}	672^{a}	758^{a}	1,240^{b}	708^{a}	702^{a}	553^{a}	911^{a}	918^{a}	1,008^{a}	17%

Clostridium	5,015^{a}	210^{bc}	414^{b}	28,146^{d}	208^{bc}	247^{bc}	7,730^{a}	184^{c}	194^{c}	264^{c}	28%
Pseudoramibacter_ Eubacterium	565^{abc}	436^{ab}	567^{ac}	967^{d}	593^{abc}	567^{abc}	320^{b}	788^{c}	707^{ac}	708^{abc}	12%
Blautia	247^{abc}	282^{a}	196^{bcd}	114^{e}	182^{bd}	203^{bd}	235^{a}	286^{abc}	319^{ac}	199^{de}	19%
Coprococcus	42^{ab}	101^{c}	38^{abd}	4,309^{e}	38^{abd}	62^{a}	20^{bd}	30^{bd}	38^{abd}	28^{d}	30%
Dorea	147^{ab}	226^{c}	117^{ab}	3,247^{d}	103^{a}	112^{a}	153^{bc}	119^{a}	148^{ab}	123^{a}	20%
[Ruminococcus]	5,475^{ab}	4,161^{a}	4,556^{ab}	2,512^{c}	6,153^{bd}	5,852^{abd}	4,510^{abd}	8,317^{de}	9,693^{e}	6,976^{ab}	15%
Ruminococcaceae	547^{a}	654^{ab}	730^{ab}	234^{c}	634^{ab}	546^{a}	625^{b}	690^{ab}	678^{ab}	970^{ab}	17%
Faecalibacterium	154^{ab}	171^{abc}	236^{cd}	67^{e}	163^{ab}	135^{a}	155^{abc}	260^{bcd}	207^{abc}	436^{d}	20%
Oscillospira	71^{a}	41^{ab}	15^{c}	721^{d}	23^{ce}	35^{be}	40^{ab}	18^{c}	19^{c}	56^{b}	27%
Ruminococcus	50^{ab}	63^{a}	60^{ab}	41^{b}	69^{a}	70^{a}	35^{ab}	75^{ab}	83^{a}	84^{ab}	23%
Dialister	2,389^{a}	2,315^{ab}	2,244^{a}	1,342^{c}	2,823^{ab}	3,201^{b}	1,745^{a}	2,838^{ab}	2,947^{ab}	3,299^{ab}	15%
Megasphaera	735^{ab}	560^{a}	509^{ac}	402^{cd}	671^{ab}	1,058^{b}	473^{a}	298^{de}	229^{e}	834^{ac}	25%
Phascolarctobacterium	8,980^{ab}	6,968^{ab}	7,434^{ab}	12,177^{a}	9,064^{ab}	10,968^{a}	5,272^{b}	9,490^{ab}	9,426^{ab}	11,752^{ab}	16%
Veillonella	44,034^{ab}	63,368^{c}	30,443^{a}	17,167^{de}	39,771^{ab}	49,181^{b}	10,300^{d}	24,758^{e}	19,449^{de}	46,731^{ab}	21%
Erysipelotrichaceae; other	0.00^{a}	0.00^{b}	0.33^{a}	0.00^{a}	0.00^{b}	0.33^{b}	0.33^{a}	0.00^{a}	0.00^{a}	0.33^{a}	173%
Sutterella	194^{a}	280^{b}	219^{abc}	127^{d}	282^{bc}	281^{bc}	73^{d}	231^{a}	223^{a}	290^{ac}	17%
Enterobacteriaceae; other	28,892^{a}	25,176^{a}	26,703^{a}	40,038^{b}	27,391^{a}	30,327^{a}	28,061^{b}	36,990^{ab}	34,887^{ab}	39,845^{a}	20%

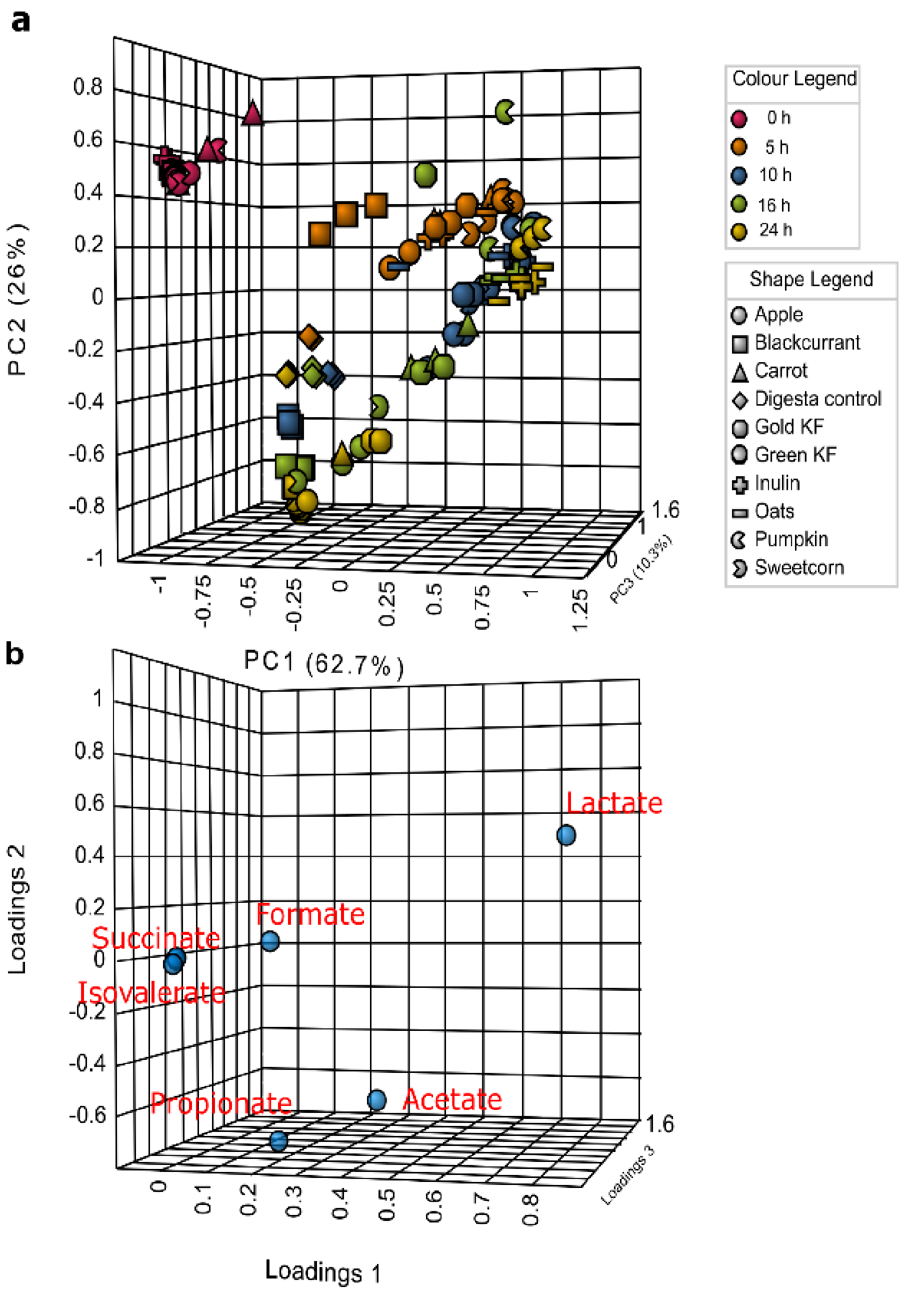

Figure S1. Principal components analysis plot (a) and the loading plot (b) visualising the significant changes in the organic acids (given in red) after fermentation of the foods and the two controls, inulin and water digesta. The colour legend denotes the time points, i.e., 0, 5, 10, 16 and 24 h of fermentation.

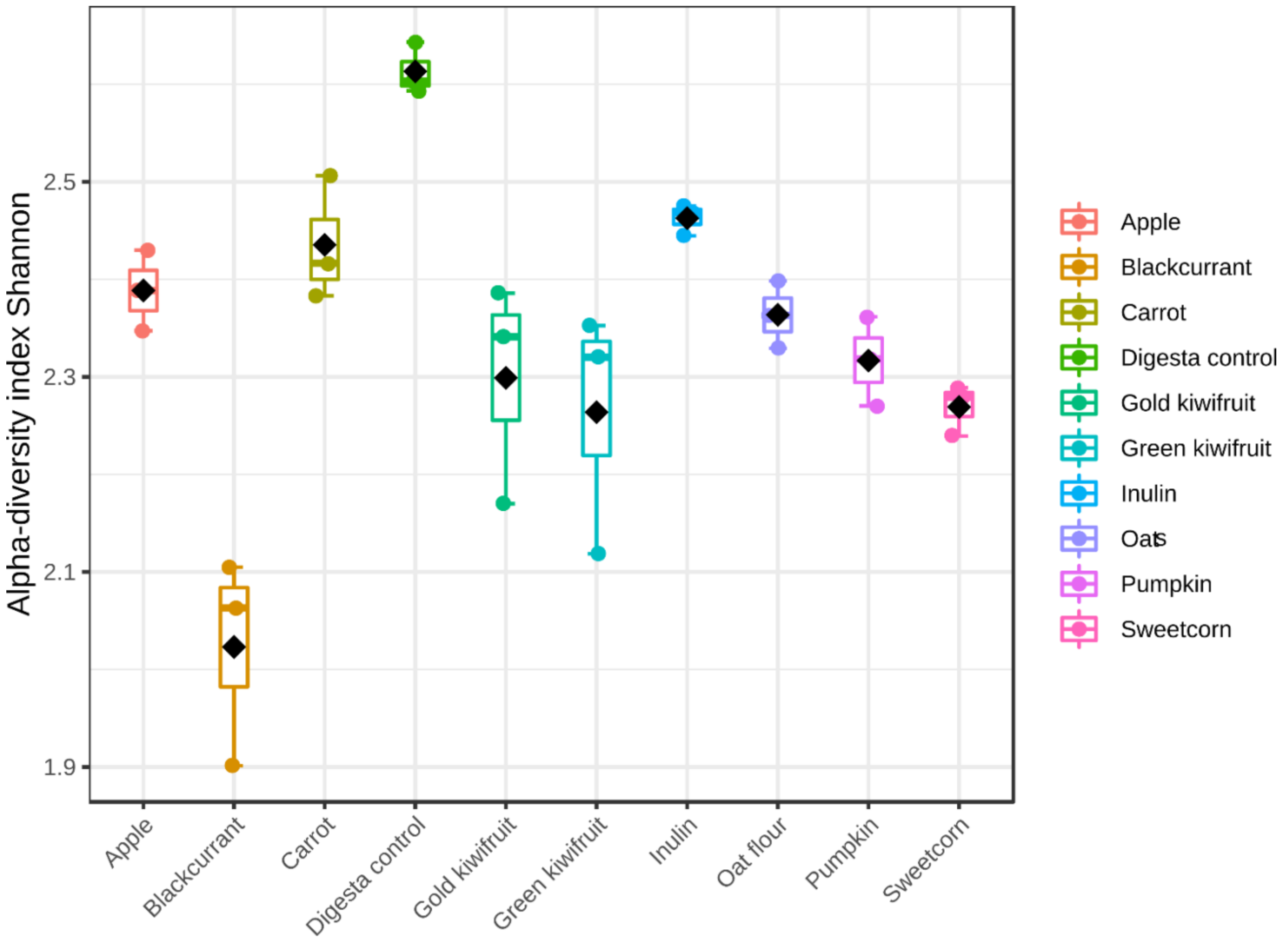

Figure S2. Shannon index depicting changes in microbiome α-diversity 10 h after fermentation of foods and the two controls, digesta control and inulin. (P = 0.003, Kruskal-Wallis Test).
